# Aqueous electrochemically-triggered atom transfer radical polymerization†

**DOI:** 10.1039/d2sc01832b

**Published:** 2022-04-27

**Authors:** Boyu Zhao, Fred Pashley-Johnson, Bryn A. Jones, Paul Wilson

**Affiliations:** University of Warwick UK p.wilson.1@warwick.ac.uk

## Abstract

Simplified electrochemical atom transfer radical polymerization (seATRP) using Cu^II^–*N*-propyl pyridineimine complexes (Cu^II^(NPPI)_2_) is reported for the first time. In aqueous solution, using oligo(ethylene glycol) methyl ether methacrylate (OEGMA), standard electrolysis conditions yield POEGMA with good control over molecular weight distribution (*Đ*_m_ < 1.35). Interestingly, the polymerizations are not under complete electrochemical control, as monomer conversion continues when electrolysis is halted. Alternatively, it is shown that the extent and rate of polymerization depends upon an initial period of electrolysis. Thus, it is proposed that seATRP using Cu^II^(NPPI)_2_ follows an electrochemically-triggered, rather than electrochemically mediated, ATRP mechanism, which distinguishes them from other Cu^II^L complexes that have been previously reported in the literature.

## Abstract 2

Simplified electrochemical atom transfer radical polymerization (seATRP) using Cu^II^-pyridineimine complexes is reported and follows a previously unreported electrochemically triggered mechanism.

## Graphical abstract

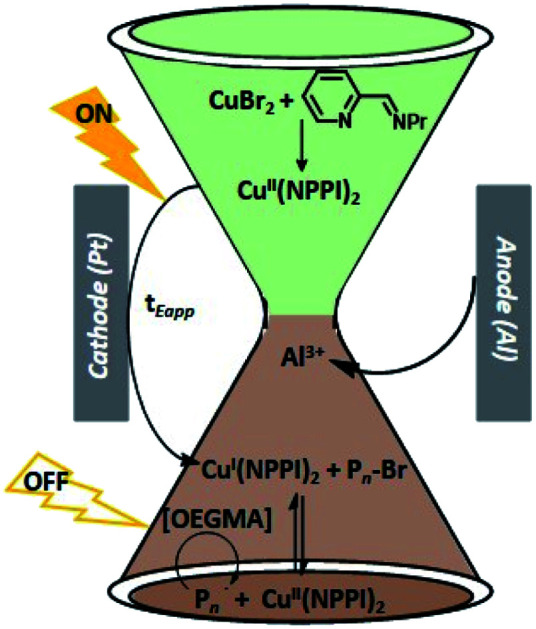

## Introduction

Electrochemical intervention in synthesis and catalysis has received renewed interest over the last 5–10 years.^1–6^ From a synthetic point of view, the use of an applied potential/current enables accurate control over the thermodynamics and/or kinetics of electron transfer processes.^7,8^ This can enhance the selectivity of chemical transformations and confer spatiotemporal control over synthetic and catalytic reactions of small and macromolecular organic molecules/polymers, amongst others.

In the context of reversible deactivation radical polymerization (RDRP) electrochemical intervention has been employed to regulate polymer synthesis through control of the dynamic equilibrium between dormant and active (radical) species which allows the overall radical concentration to be accurately controlled.^9–11,53^ In atom transfer radical polymerization (ATRP)^12,13^ the equilibrium (*K*_ATRP_) is between a dormant alkyl (R–X) or macromolecular (P_*n*_–X) halide and propagating radicals (R˙/
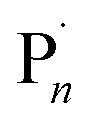
) which undergo reversible redox reactions with transition metal complexes.

In 2011, Matyjaszewski and co-workers showed that the redox nature of the Cu-mediated ATRP mechanism could lend itself to electrochemical manipulation and control.^10^ The active, yet oxidatively labile Cu^I^L complex was formed *in situ* when a reducing potential (*E*_app_) was applied at the working electrode (WE) to induce a one electron reduction of an inactive Cu^II^L precursor. Activation of the dormant species (R–X/P_*n*_–X) in the reaction media then generated the radical species (R˙/
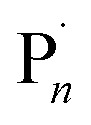
), and the Cu-complex in a higher oxidation state (X–Cu^II^L). Well controlled polymerization of methyl acrylate was reported suggesting that the deactivation step of the equilibrium, between the propagating radical 
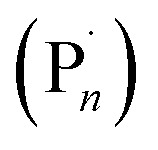
 and X–Cu^II^L, reforming the dormant species (P_*n*_–X) and Cu^I^L respectively, was not perturbed by the electrochemical intervention. In fact, it was shown that by switching the *E*_app_ at the WE to an oxidizing potential the polymerization could be completely switched off, conferring high fidelity on-off spatiotemporal control over polymer synthesis in solution.

In the 10 years since this discovery, eATRP has been employed for the synthesis of polymers with a variety of compositions and architectures including block copolymers, bioconjugates, star and graft (co)polymers.^14–22^ It is compatible with aqueous^23,24^ and organic^10^ media whilst heterogeneous systems such mini-emulsion^25–28^ and surface-initiated (si-eATRP)^29–32^ polymerizations have also been reported. Furthermore, the complex reaction set-up, initially involving a 3-electrode divided electrochemical cell, has been simplified by the use of sacrificial counter electrodes (typically Al-wire), enabling undivided cells to be used in either 3-electrode (potential controlled) or 2-electrode (current controlled) configurations giving rise to simplified electrochemical atom transfer radical polymerisaiton (seATRP).^33^ This development is significant as it enables the chemistry to be performed using commercial, standardized hardware.^24^

The most widely studied systems for aqueous eATRP employ Cu^II^X salts with tetradentate ligands *tris*(2-(dimethylamino)ethyl)amine (Me_6_-Tren)^10,34,35^ or *tris*(2-pyridylmethyl)amine (TPMA).^33,36,37^ They form more active complexes, having high *K*_ATRP_ values.^38^ The ligands stabilize Cu^II^ more than Cu^I^ with cyclic voltammetry (CV) indicating that Cu^I^Me_6_-Tren and Cu^I^TPMA are strongly reducing complexes, leading to fast activation (*k*_act_) of R–X/P_*n*_–X.^39,40^ The *k*_act_ (and *K*_ATRP_) can increase by orders of magnitude when aqueous media is employed, which in the absence of appropriate conditions and/or external control of active catalyst generation, can result in high radical concentrations which has a detrimental effect on the polymerization.^41,42^ A great deal of discovery and optimization, of which eATRP is one example, has resulted in the development of efficient, well controlled aqueous ATRP reactions using these highly active complexes.^9,43^

Prior to this, less active complexes composed of bidentate ligands such as bipyridine (bpy) and *N*-alkyl pyridine imines (NAPI) were more suitable for aqueous ATRP.^44–49^ They stabilize Cu^I^ more than Cu^II^, form less reducing Cu^I^ complexes and have lower *k*_act_ and *K*_ATRP_ leading to lower radical concentrations. On one hand, this means that larger catalyst concentrations are required to mediate well controlled ATRP. On the other hand, it can also be beneficial for polymerizations carried out in aqueous media wherein increased *k*_act_ and *K*_ATRP_ can lead to higher radical concentrations when more active complexes are employed. For example, 20 years ago, Haddleton and Perrier described in detail the efficient, well controlled polymerization of oligo(ethylene glycol) methyl ether methacrylate (OEGMA) using Cu^I^(NAPI)_2_ complexes in water.^44–46^ The rates of reaction and control over the polymerization were optimized with respect to [Cu^I^(NAPI)_2_]/[Cu^II^(NAPI)_2_] which was controlled from the outset by using known amounts of Cu^I^Br and Cu^II^Br_2_ to form a mixed complex system. To accurately achieve the target [Cu^I^(NAPI)_2_]/[Cu^II^(NAPI)_2_] ratio's careful handling of oxidatively labile Cu^I^ complexes and thoroughly deoxygenated reaction conditions were required. Looking back at this work, we considered the possibility of controlling [Cu^I^(NAPI)_2_]/[Cu^II^(NAPI)_2_] electrochemically, thus avoiding the need to handle the oxidatively labile Cu^I^ complexes. There are currently no reports of eATRP using Cu(NAPI)_2_ complexes, in either organic or aqueous media in the literature. We were inspired to investigate these complexes with a view to mediate eATRP at less reducing potentials and currents. Long term, we hope this will help to overcome some of the initial limitations associated with oxygen reduction (at more reducing potentials) in our related work in scanning electrochemical probe directed eATRP.^50^

To this end, herein we report for the first time the use of the *N*-propyl pyridineimine (NPPI) ligand to form Cu^II^(NPPI)_2_ complexes for eATRP of OEGMA_300_. Well controlled polymerization (*Đ*_m_ ≈ 1.30) is possible and initial investigations into the mechanism suggest that an alternative electrochemically-triggered process is prevalent for these less-active copper complexes.

## Results and discussion

Comparative CV of Cu^II^L complexes; Cu^II^TPMA, Cu^II^Me_6_Tren and Cu^II^(NPPI)_2_ were initially performed in solutions of the reaction mixture (10% (v/v) OEGMA_300_ in H_2_O) in the absence and presence of the initiator, hydroxyethyl-2-bromoisobutyrate (HEBiB) (Fig. S1–S3†). In the absence of HEBiB, each complex exhibited the [Cu^II^L]/[Cu^I^L] redox process and as expected the standard reduction potential (*E*^*θ*^ ≈ *E*_1/2_ = *E*_pc_ + *E*_pa_/2) shifted to less reducing potentials (*vs.* Ag/AgCl) going from Cu^II^Me_6_Tren (*E*_1/2_ = −0.40 V) to Cu^II^TPMA (*E*_1/2_ = −0.21 V) to Cu^II^(NPPI)_2_ (*E*_1/2_ = +0.02 V) respectively. In the presence of HEBiB the voltammograms of the Cu^II^Me_6_Tren and Cu^II^TPMA complexes show a coupled increase in the cathodic current intensity (*E*_pc_) and decrease in the anodic current intensity (*E*_pa_). This is indicative of electrochemical reduction of Cu^II^L to Cu^I^L followed by fast activation of HEBiB by the Cu^I^L on the timescale of the CV (0.1 V s^−1^). In the case of Cu^II^(NPPI)_2_ the coupled change in *E*_pc_ and *E*_pa_ was not observed. The currents decrease in both the cathodic and anodic scan suggesting that although the presence of HEBiB has an effect on the kinetics of electron transfer, the activation of HEBiB by Cu^I^(NPPI)_2_ is slow on the timescale of the CV. These results are in agreement with the literature that suggests that with respect to *k*_act_, complexes with Me_6_Tren > TPMA ≫ NPPI.^35,38^

Potentiostatic seATRP reactions using each complex were performed in undivided cells using an IKA ElectraSyn device. A commercial Pt-coated electrode (IKA) was employed as the cathode (WE), the anode (CE) was Al-wire and the reference electrode (RE) was Ag^+^/AgCl. For the bidentate NPPI ligand a ratio of [OEGMA_300_] : [HEBiB] : [CuBr_2_] : [NPPI] = [20] : [1] : [0.5] : [1.25] was used. When *E*_app_ = *E*_1/2_ = +0.02 V the resistance in the system was too high preventing the IKA ElectraSyn from operating. However, when an overpotential of 60 mV was applied (*E*_app_ = −0.04 V) polymerization was complete within 2 h yielding POEGMA_300_ with *M*_n,SEC_ = 9200 g mol^−1^ and *Đ*_m_ = 1.31 (Table 1, entry 1, Fig. S4†).

**Table 1:** seATRP of OEGMA_300_ in H_2_O. [OEGMA_300_] : [HEBiB] : [CuBr_2_] : [NPPI] = [20] : [1] : [0.5] : [1.25]; room temperature^a^

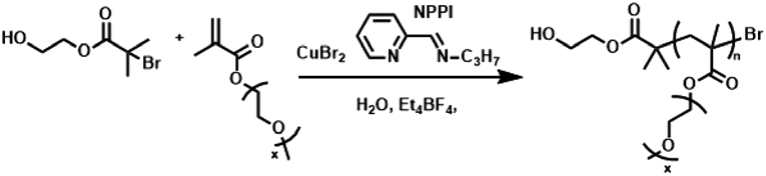						
Entry	*E*_app_/V	Time/h	Conv^k^	*M*_n,th_ l/g mol^−1^	*M*_n,SEC_ m/g mol^−1^	*Đ*_m_ m
1	−0.04	2	100%	6211	9200	1.31
2	−0.08	2	96%	5971	11 000	1.30
3	−0.12	2	96%	6211	12 800	1.29
4	−0.16	2	99%	6211	10 200	1.32
5^b^	−0.16	2	100%	6211	10 000	1.28
6^c^	−0.16	2	100%	6211	9000	1.26
7^d^	−0.16	2	96%	5971	18 200	1.50
8^e^	−0.16	2	67%	4231	21 800	1.98
9^f^	−0.16	2	96%	9811	14 600	1.30
10^g^	−0.16	6.5	67%	14 951	14 900	1.23
11^h^	−0.16	2	98%	3151	7800	1.31
12^i^	−0.16	2	90%	11 011	14 000	1.33
13^j^	−0.16	2	95%	23 011	21 500	1.33

a [Cu^II^Br_2_] = 8.8 mM.

b [OEGMA_300_] = 20% v/v.

c [OEGMA_300_] = 30% v/v.

d [Cu^II^Br_2_] = 4.4 mM.

e [Cu^II^Br_2_] = 2.2 mM.

f OEGMA_500_ used.

g OEGMA_1100_ used.

h [M]/[I] = 10.

i [M]/[I] = 40.

j [M]/[I] = 80.

k Determined *via*^1^H NMR of reaction samples performed in D_2_O.

l *M*_n,th_ = [(conv./100 × DP_*n*,th_) *x* 300/500(e)/1100(f)] + 211.

m From THF SEC.

When Me_6_Tren and TPMA were employed as ligands ([OEGMA_300_] : [HEBiB] : [CuBr_2_] : [L] = [20] : [1] : [0.5] : [0.6]), *E*_app_ = *E*_1/2_ was sufficient for the ElectraSyn to operate. In both cases, conversions were limited to <80% after 4 h and the control over the polymerization was poor (*Đ*_m_ > 4, Table S1†). This is likely due to the stoichiometry of Cu^II^Br_2_ employed which equates to [Cu^II^Br_2_] = 8.8 mM. Although, this is suitable for the less active Cu^II^/NPPI system, it is much higher than is required for the so-called highly active complexes leading to higher than necessary [Cu^I^L] and [R˙/
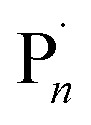
] due to rapid over activation when *E*_app_ = *E*_1/2_ which ultimately compromises the outcome of the polymerization.

Incrementally increasing the overpotential by 40 mV had little effect on the rate of the polymerization with conversions remaining high (>95%), and control being retained with *Đ*_m_ ≈ 1.30 (Table 1, entries 2–4, Fig. S5–S7†). The *E*_app_ was then fixed at −0.16 V and [OEGMA_300_] was increased to 20% and 30% v/v respectively (Table 1, entries 5–6). There was no significant change in the control over the polymerization with low dispersities (*Đ*_m_ < 1.30) obtained (Fig. S8 and S9†). Kinetic analysis showed that quantitative conversions were obtained with 2 h. However, the semi-log plot showed that whilst the pseudo first order kinetics were observed at [OEGMA_300_] = 10% v/v, distinct deviations were apparent at the higher concentrations (Fig. S10†). The observed increase in rate throughout the reaction is in agreement with Haddleton and Perrier who attributed it to water and monomer completing with the ligand for coordination at the Cu centre thus affecting the Cu^II^/Cu^I^ equilibrium.^44^ With this in mind, the remaining reactions were performed at [OEGMA_300_] = 10% v/v.

Decreasing the [Cu] from 8.8 mM to 4.4 mM and 2.2 mM had a detrimental effect on the control over the polymerization (*Đ*_m_ > 1.50, Table 1, entries 7–8). Increasing the length of the OEGMA monomer using OEGMA_500_ and OEGMA_1100_ had little effect on the control over the polymerization, with low dispersities retained (*Đ*_m_ < 1.30, Fig. S11 and S12†), though the rate of polymerization for OEGMA_1100_ was slower than OEGMA_300/500_ reaching 67% within 6.5 h (Table 1, entries 9–10).

**Fig. 1:**
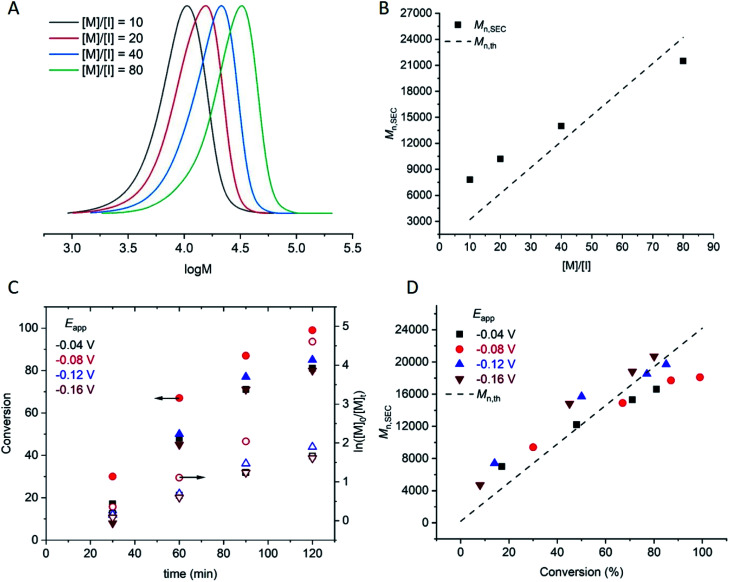
For seATRP of [OEGMA_480_] : [HEBiB] : [CuBr_2_] : [NPPI] = [M] : [1] : [0.5] : [1.25]; (A) SEC in THF showing a shift in molecular weight distributions as for [M] = 10, *M*_n_ = 7800 g mol^−1^, *Đ*_m_ = 1.31; [M] = 20, *M*_n_ = 10 200 g mol^−1^, *Đ*_m_ = 1.32, [M] = 40, *M*_n_ = 14 000 g mol^−1^, *Đ*_m_ = 1.33, [M] = 80, *M*_n_ = 21 500 g mol^−1^, *Đ*_m_ = 1.33. (B) Plot of *M*_n,SEC_ as a function of [M]/[I] for [M] = 10, 20, 40, 80, [I] = 1, (*E*_app_ = −0.16 V). (C) Conversion and pseudo first order kinetic plots as a function of *E*_app_. (D) Evolution of the *M*_n,SEC_ with conversion during polymerizations performed at different *E*_app_ ([M]/[I] = 80).

Under the conditions established above (*E*_app_ = −0.16 V; [M] = 10% v/v), the DP_*n*,th_ was varied such that [M] : [I] = [10]/[20]/[40]/[80] : [1]. The polymerizations reached 90–98% conversion within 2 h, proceeding with good control over *M*_n_ and dispersity (*Đ*_m_ < 1.35; Table 1, entries 4, 11–13). An overlay of the SEC chromatograms shows the expected shift in the narrow molecular weight distributions to higher molecular weights as a function of [M]/[I] (Fig. 1A). A plot of *M*_n,SEC_*vs.* [M]/[I] indicates a linear increase in *M*_n,SEC_ as a function of [M]/[I], with slight deviations in *M*_n,SEC_ and *M*_n,th_ converging as [M]/[I] increased (Fig. 1B).

Kinetic analyses of the polymerizations performed with [M] : [I] = [80] : [1] revealed that the apparent rate constant for propagation was *k*^app^_p_ = 0.0167 min^−1^ at *E*_app_ = −0.04 V (Fig. 1C). Initially, more reducing potentials (*E*_app_ = −0.08 V) resulted in a small increase in the rate of polymerization (*k*^app^_p_ = 0.0456 min^−1^). However, at higher overpotentials (*E*_app_ = −0.12 V, −0.16 V), the rate decreased back to *k*^app^_p_ = 0.0201 min^−1^ and 0.0174 min^−1^ respectively. At these potentials, *E*_app_ is close to *E*_pc_ at which point the reduction of Cu^II^/L to Cu^I^/L is not governed by the electrode potential and is limited by the rate of diffusion of accumulated Cu/L species to and from the electrode surface to and from the bulk. Irrespective of *E*_app_, a linear increase in *M*_n,SEC_ as a function of conversion was observed with good agreement with the theoretical molecular weight (*M*_n,th_) (Fig. 1D).

**Fig. 2:**
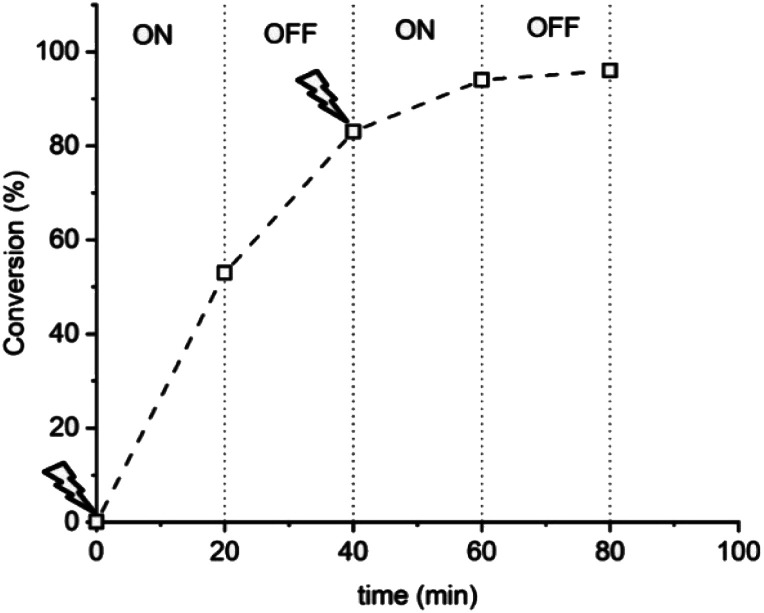
Conversion *vs.* time plot demonstrating a lack of temporal control afforded by seATRP with [OEGMA_300_] : [HEBiB] : [CuBr_2_] : [NPPI] = [20] : [1] : [0.5] : [1.25] : [0.15]; *E*_app_ = −0.16 V.

A hallmark of eATRP is the temporal control conferred by switching the potential/current on and off. At reducing potentials Cu^II^/L is reduced to Cu^I^/L leading to activation of dormant chains which can undergo propagation and subsequent deactivation events *via* the proposed ATRP mechanism. If the potential is switched off, or an oxidising potential is applied, reduction of Cu^II^/L nolonger occurs so activation of the dormant chains stops and the polymerization is halted. Conducting this experiment using [OEGMA_300_] : [HEBiB] : [CuBr_2_] : [NPPI] = [20] : [1] : [0.5] : [1.25] and applying *E*_app_ = −0.16 V ([M] = 10% v/v), conversion reached >50% within 20 min (Fig. 2). The potential was then switched off (*E*_app_ = 0 V) and stirring was continued for a further 20 min, after which conversion unexpectedly increased to >80%. The polymerization reached >90% conversion through an additional ‘on’ (20 min) and ‘off’ (20 min) cycle, indicating that Cu^II^(NPPI)_2_ lacked the temporal control associated with eATRP. The lack of temporal control with this less active catalyst system is in agreement with reported differences in temporal control related to catalyst activity observed in photo-ATRP.^51^

Unlike in eATRP reactions using Cu^II^(Me_6_Tren) and Cu^II^(TPMA) complexes, it was observed that the reaction solutions containing Cu^II^(NPPI)_2_ changed colour, from green to brown, during electrolysis (Fig. S14†). The brown colour resembled that reported in early aqueous ATRP using Cu^I^(NAPI)_2_ complexes.^44–46^ Thus, it was hypothesized that the overpotentials applied and the less activating nature of the Cu^I^(NPPI)_2_ complex, resulted in accumulation of stable Cu^I^(NPPI)_2_ in the reaction media which was capable of continuing to mediated the polymerization of OEGMA when *E*_app_ was removed.

To explore this hypothesis, the temporal control experiment was repeated using [OEGMA_300_] : [HEBiB] : [CuBr_2_] : [NPPI] = [20] : [1] : [0.5] : [1.25]. After the electrolysis period (*E*_app_ = −0.08 V; *t*_*E*_app__ = 30 min), the reaction solution was brown, indicative of Cu^I^(NPPI)_2_ accumulation, and conversion had reached 33% (Fig. S15A†). Concurrently, electrolysis was stopped and sparging with compressed air was commenced to rapidly introduce O_2_ into the reaction solution to stop the polymerization. The solution quickly changed colour from brown to green, indicative of oxidation of Cu^I^(NPPI)_2_ to Cu^II^(NPPI)_2_ and no further conversion was observed (Fig. S15B†). In an attempt to re-initiate the polymerization, the reaction solution was sparged for second time, this time with N_2_ to displace the O_2_ previously added to the solution, prior to a second period of electrolysis. Pleasingly, re-initiation was observed (*E*_app_ = −0.08 V; *t*_*E*_app__ = 30 min) with the polymerization reaching 64% conversion (Fig. S15C†), yielding POEGMA_300_ with *M*_n,SEC_ = 9800 g mol^−1^ and *Đ*_m_ = 1.25 (Fig. S16†) which is comparable to the POEGMA_300_ obtain during the constant electrolysis reactions.

**Fig. 3:**
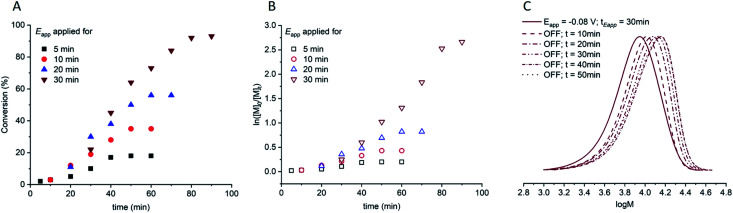
For triggered seATRP of [OEGMA_300_] : [HEBiB] : [CuBr_2_] : [NPPI] = [20] : [1] : [0.5] : [1.25]; (A) conversion *vs.* time plot for polymerizations with different *t*_*E*_app__. (B) Pseudo first order kinetic plots for polymerizations for *t*_*E*_app__ = 5 min, *k*^app^_p_ = 0.0028 min^−1^; *t*_*E*_app__ = 10 min, *k*^app^_p_ = 0.0046 min^−1^; *t*_*E*_app__ = 20 min, *k*^app^_p_ = 0.0218 min^−1^; *t*_*E*_app__ = 30 min, *k*^app^_p_ = 0.0425 min^−1^. (C) SEC in THF showing the evolution of the molecular weight distribution after electrolysis (*E*_app_ = −0.08 V, *t*_*E*_app__ = 30 min, solid line) and at 10 minutes intervals after the potential was removed (*E*_app_ = 0 V, dashed lines, final *M*_n,SEC_ = 9300 g mol^−1^, *Đ*_m_ = 1.33).

A series of experiments was then performed in which reaction solutions ([OEGMA_300_] : [HEBiB] : [CuBr_2_] : [NPPI] = [20] : [1] : [0.5] : [1.25]) were electrolyzed at constant potential (*E*_app_ = −0.08 V) for increasing periods of time (*t*_*E*_app__ = 5, 10, 20, 30 min) before the potential was removed (*E*_app_ = 0 V). Samples were taken for analysis after electrolysis and at regular intervals after the potential was removed. Increasing the initial electrolysis times led to an increase in initial conversion from 2% (*E*_app_ = −0.08 V; *t*_*E*_app__ = 5 min) to 22% (*E*_app_ = −0.08 V; *t*_*E*_app__ = 30 min). In all experiments monomer conversion continued upon removal of *E*_app_ (Fig. 3A). At shorter electrolysis times, conversion continued up to a total reaction time of 60 min resulting in final conversions of 18% and 35% when *t*_*E*_app__ = 5 and 10 min respectively. Increasing the initial electrolysis time to 20 min yielded initial conversions of 11% with monomer conversion continuing thereafter to reach a final conversion of 56% after a total reaction time of 70 min. When the reaction solution was electrolyzed for 30 min monomer conversion continued for a total reaction time of 90 min, reaching 93% conversion. Kinetic analysis of these reactions revealed that the rate of the reaction also increased from *k*^app^_p_ = 0.0028 min^−1^ when *t*_*E*_app__ = 5 min to *k*^app^_p_ = 0.0425 min^−1^ when *t*_*E*_app__ = 30 min (Fig. 3B). An overlay of the SEC chromatograms shows that the polymerization continues after the initial electrolysis period with the molecular weight distributions shifting to higher molecular weights as a function of time (Fig. 3C). The final polymer obtained (*E*_app_ = −0.08 V; *t*_*E*_app__ = 30 min) was comparable to the polymer obtained by uninterrupted electrolysis (Table 1; entry 2) with *M*_n,SEC_ = 9300 g mol^−1^ and *Đ*_m_ = 1.33.

**Fig. 4:**
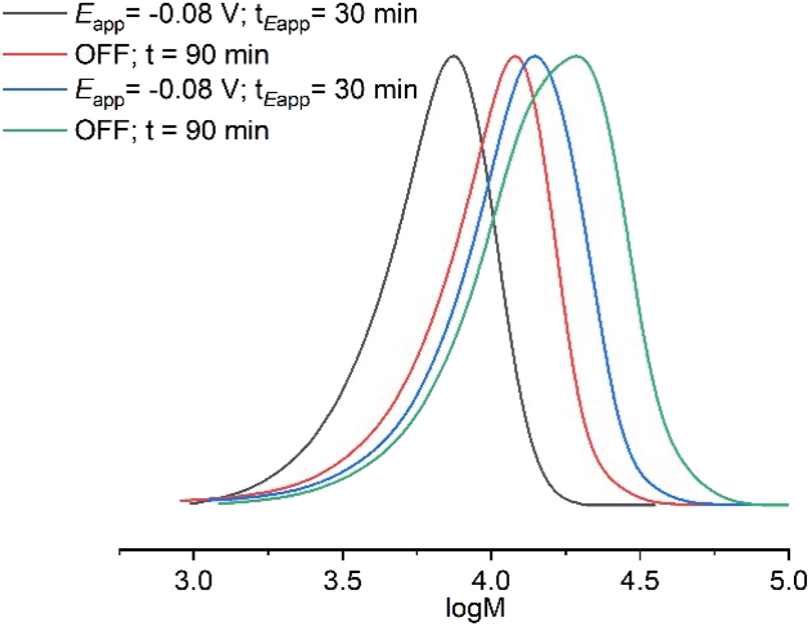
SEC in THF showing the evolution of the molecular weight distribution during electrochemically triggered eATRP of OEGMA_300_ ([OEGMA_300_] : [HEBiB] : [CuBr_2_] : [NPPI] = [20] : [1] : [0.5] : [1.25]; *E*_app_ = −0.08 V; *t*_*E*_app__ = 30 min) and chain extension ([OEGMA_300_] : [POEGMA_300_-Br] = [20] : [1]; *E*_app_ = −0.08 V; *t*_*E*_app__ = 30 min) (Table 2).

Quantification of the end group fidelity using conventional ^1^H NMR analysis was not possible as poly(methacrylates) do not contain an ω-methine proton to integrate against signals at the α-chain end. To exemplify end group fidelity, a chain extension experiment was performed. Homopolymerization of OEGMA_300_ was performed using [OEGMA_300_] : [HEBiB] : [CuBr_2_] : [NPPI] = [20] : [1] : [0.5] : [1.25] (*E*_app_ = −0.08 V). After electrolysis for 30 min (Table 2, entry 1) and stirring at room temperature in the absence of electrolysis for an additional 90 min near quantitative conversion was obtained (Table 2, entry 2, Fig. S17†). At this point a second aliquot of OEGMA_300_ (1 mL, [OEGMA_300_] : [POEGMA_300_-Br] = [20] : [1]) was added and electrolysis was again applied (*E*_app_ = −0.08 V) for 30 min (Table 2, entry 3) followed by stirring in the absence of electrolysis for an additional 90 min reaching a final conversion of 81% (Table 2, entry 4). A clear shift in the molecular weight distribution was evident *via* SEC analysis (Fig. 4). The molecular weight of the final POEGMA_300_ obtained (*M*_n,SEC_ = 16 600 g mol^−1^) was in reasonable agreement to the theoretical molecular weight (*M*_n,th_ = 12 200 g mol^−1^) relative to the homopolymerizations performed. Although this result exemplifies good chain-end fidelity, there is scope for optimization based on a gradual increase in tailing to low molecular weight, which increased during the course of the reaction resulting in a gradual increase in dispersity (*Đ*_m_ = 1.27–1.45, Table 2).

**Table 2:** Electrochemically triggered seATRP of OEGMA_300_ in H_2_O. [OEGMA_300_] : [HEBiB] : [CuBr_2_] : [NPPI] = [20] : [1] : [0.5] : [1.25]; followed by *in situ* chain extension using OEGMA_300_ (20 eq.); *E*_app_ = −0.08 V; *t*_*E*_app__ = 30 min; room temperature^a^

Entry	*E*_app_/V	Time/min (*t*_total_)	Conv^b^	*M*_n,th_/g mol^−1^	*M*_n,SEC_ e/g mol^−1^	*Đ*_m_ e
1	−0.08	30 (30)	33%	2191^c^	7300	1.26
2	—	90 (120)	>99%	6211^c^	11 300	1.31
3	−0.08	30 (150)	57%	9631^d^	13 800	1.34
4	—	90 (240)	81%	11 071^d^	16 600	1.45
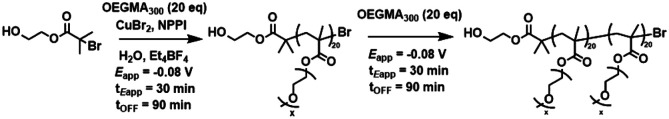						

a [Cu^II^Br_2_] = 8.8 mM.

b Determined *via*^1^H NMR of reaction samples performed in D_2_O.

c *M*_n,th_ = [(conv./100 × DP_*n*,th_) × 300] + 211.

d *M*_n,th_ = [(conv./100 × DP_*n*,th_) × 300] + 6211.

e From THF SEC.

The electrochemically triggered reaction conditions were also compatible with polymerizations of OEGMA_300_ targeting higher molecular weights. When DP_*n*,th_ = 200, the reaction solution was electrolyzed for 30 min (*E*_app_ = −0.08 V) leading to 13% conversion. The reaction continued in the absence of electrolysis for an additional 90 min, reaching 89% conversion (Fig. S18†) furnishing POEGMA_300_ with relatively low dispersity (*Đ*_m_ = 1.32, Fig. S19†). To expand the monomer scope, 2-*N*-morpholinoethyl methacrylate (DP_*n*,th_ = 200) was electrolyzed for 30 min (*E*_app_ = −0.08 V) resulting in 40% conversion. The reaction was allowed to continue in the absence of electrolysis for an additional 90 min, reaching 65% conversion (Fig. S20 and S21†).

**Fig. 5:**
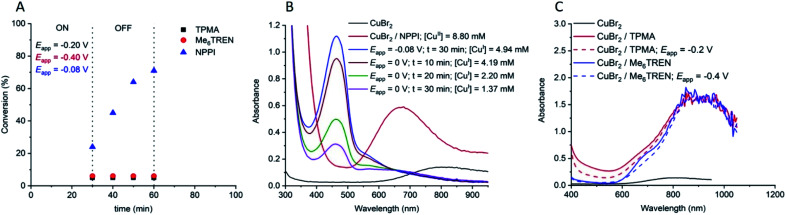
(A) Conversion *vs.* time plot for seATRP of [OEGMA_300_] : [HEBiB] : [CuBr_2_] : [L] = [20] : [1] : [0.5] : [L]. For NPPI [L] = [1.25] and for TPMA and Me_6_Tren [L] = [0.6] (*t*_*E*_app__ = 30 min). (B) UV-vis traces showing the reduction of Cu^II^(NPPI)_2_ to Cu^I^(NPPI)_2_ and the change in [Cu^I^(NPPI)_2_] during the triggered seATRP of [OEGMA_300_] : [HEBiB] : [CuBr_2_] : [NPPI] = [20] : [1] : [0.5] : [1.25]. Using *ε* = 1359 M^−1^ cm^−1^ [Cu^I^(NPPI)_2_] was quantified at each point (see ESI† for calibration and calculation details). (C) UV-vis traces of Cu^II^(TPMA) and Cu^II^(Me_6_Tren) before and after electrolysis. *E*_app,TPMA_ = −0.20 V, *E*_app,Me6Tren_ = −0.40 V, *t*_*E*_app__ = 30 min.

To explore the mechanism, the polymerization using [OEGMA_300_] : [HEBiB] : [CuBr_2_] : [NPPI] = [20] : [1] : [0.5] : [1.25] (*t*_*E*_app__ = 30 min; *E*_app_ = −0.08 V) was repeated and the electrochemical reduction of Cu^II^(NPPI)_2_ to Cu^I^(NPPI)_2_ followed by UV-vis spectroscopy (Fig. 5). Prior to electrolysis the reaction solution was green and the characteristic Cu^II^(NPPI)_2_ absorbance band was present at *λ* = 670 nm, assigned to the d–d transitions of the Cu^II^ centre. After electrolysis the reaction solution was brown in colour, qualitatively confirming the reduction of Cu^II^(NPPI)_2_ to Cu^I^(NPPI)_2_. UV-vis of the reaction solution immediately after electrolysis showed disappearance of the absorbance band at *λ* = 670 nm and appearance of a new, strong absorbance band at *λ* = 465 nm, confirming reduction of Cu^II^(NPPI)_2_ to Cu^I^(NPPI)_2_. The absorbance at *λ* = 465 nm was assigned to MLCT between the Cu^I^ centre and the π* of the surrounding NPPI ligands, as reported for other bipyridyl and/or diimine based complexes of Cu^I^.^52^

In order to quantify the concentration of Cu^I^(NPPI)_2_ present after electrolysis, a calibration plot of Cu^I^(NPPI)_2_ was used to determine the molar extinction coefficient of Cu^I^(NPPI)_2_ (*ε* = 1359 M^−1^ cm^−1^, Fig. S22†). Prior to electrolysis, the concentration of Cu^II^(NPPI)_2_ in the reaction solution was 8.8 mM. After electrolysis for 30 min, conversion reached 24% (Fig. 5A) and [Cu^I^(NPPI)_2_] was measured and found to be 4.94 mM (Fig. 5B). The reaction was again allowed to continue in the absence of an applied potential (*E*_app_ = 0 V). Though the polymerization continued, the colour of the reaction solution gradually changed from brown back to green over the course of the reaction. Further UV-vis analysis of the reaction solution allowed [Cu^I^(NPPI)_2_] to be followed, revealing a steady decrease over time eventually reaching 1.37 mM after 30 min at which point the reaction had reached 72% conversion.

Identical analyses were performed during polymerization of OEGMA_300_ using Cu^II^TPMA and Cu^II^Me_6_-Tren. Due to the tetradentate nature of TPMA and ME_6_-Tren, the reaction solutions were composed of [OEGMA_300_] : [HEBiB] : [CuBr_2_] : [TPMA/Me_6_-Tren] = [20] : [1] : [0.5] : [0.6]. Both Cu^II^TPMA and Cu^II^Me_6_-Tren produced blue solutions prior to electrolysis. Qualitatively, no colour change was observed upon electrolysis. The *E*_app_ employed was selected based on the *E*_1/2_ (−0.2 V, TPMA; −0.4 V Me_6_-Tren; Fig. S1 and S2†) and electrolysis was initially performed for 30 min before the potential was removed and stirring continued at room temperature. The progress of the reactions was followed by ^1^H NMR revealing ∼5% conversion after the initial period of electrolysis. UV-vis analysis showed very little change in the absorbance spectra of each complex (Fig. 5C) and unlike the Cu^II^(NPPI)_2_ system, no further conversion of monomer to polymer was observed when the reaction was continued for 30 min at *E*_app_ = 0 V (Fig. 5A). This is perhaps unsurprising considering the relative activity of the Cu^I^TPMA and Cu^I^Me_6_-Tren complexes relative to Cu^I^(NPPI)_2_. Thus we repeated the reaction using bipyridine (bipy) to form Cu^I^(bipy)_2_*in situ* which has intermediate activity relative to the highly active complexes derived from Me_6_-Tren/TPMA and the less active complex derived from NPPI. Similar to Cu^II^(NPPI)_2_, initial conversion in the presence of Cu^II^(bipy)_2_ increased with increasing electrolysis time (*E*_app_ = −0.08 V; *t*_*E*_app__ = 10–30 min). However, in the absence of electrolysis polymerization was only maintained for 10–20 min reaching only moderate final conversions (< 65%, *E*_app_ = −0.08 V; *t*_*E*_app__ = 30 min, Fig. S23†). This suggests that the ability to conduct electrochemically triggered eATRP is directly related to the activity (*k*_act_ and *K*_ATRP_) of the Cu-complex employed.

Overall, these results consolidate the hypothesis that the less activating nature of the Cu^I^(NPPI)_2_ complex, and its stability in water results in its accumulation in the reaction media. The accumulated Cu^I^(NPPI)_2_ is then capable of mediating the polymerization of OEGMA when *E*_app_ was removed. We therefore propose that Cu complexes containing pyridine-imine ligands (Cu^II^(NAPI)_2_) follow an electrochemically-triggered, rather than electrochemically mediated, ATRP mechanism wherein *E*_app_ is only required in order to generate the required [Cu^I^(NAPI)_2_] to initiate and maintain the polymerization reaction.

**Fig. 6:**
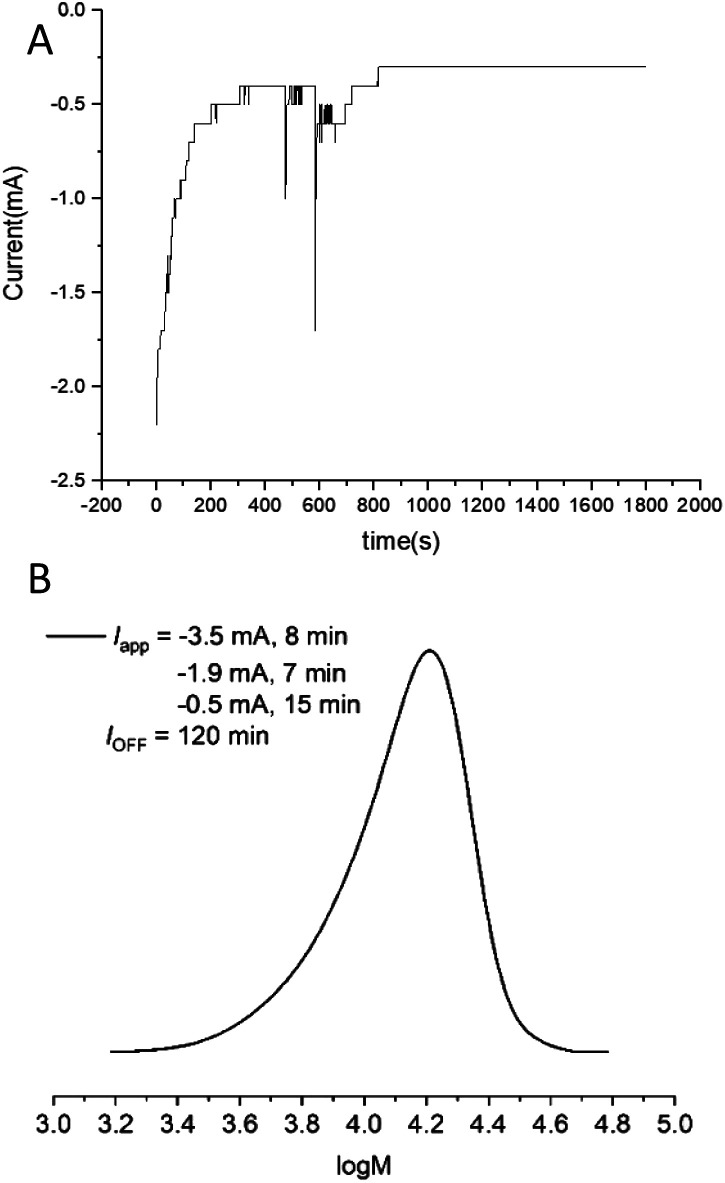
(A) *I vs. t* plot for the electrochemically triggered seATRP reaction of OEGMA_300_ performed under potentiostatic conditions [OEGMA_300_] : [HEBiB] : [CuBr_2_] : [NPPI] = [20] : [1] : [0.5] : [1.25]; *E*_app_ = −0.08 V; *t*_*E*_app__ = 30 min, *Q* = 0.78 C). (B) Molecular weight analysis of the final polymer formed from the same reaction performed using step-wise current profile min (*I*_app_ = −3.5 mA, 8 min; −1.9 mA, 7 min; −0.5 mA, 15 min). SEC in THF *M*_n,SEC_ = 11 600 g mol^−1^, *Đ*_m_ = 1.25.

Finally, to simplify the reaction set up further, current *vs.* time (*I vs. t*) graphs obtained from reactions performed under potentiostatic conditions (Fig. 6A) were used to design a step-wise current profile to enable the electrochemically triggered polymerizations to be performed using a 2-electrode current controlled configuration. Using [OEGMA_300_] : [HEBiB] : [CuBr_2_] : [NPPI] = [20] : [1] : [0.5] : [1.25], a 3-step current profile was initially applied over 30 min (*I*_app_ = −3.5 mA, 8 min; −1.9 mA, 7 min; −0.5 mA, 15 min) resulting in 12% conversion. At this point the reaction continued in the absence of electrolysis for a further 120 min reaching 80% conversion (Fig. S24†) with comparable control (*M*_n,SEC_ = 11 600 g mol^−1^; *Đ*_m_ = 1.25, Fig. 6B) to the polymerizations performed with a potentiostatic trigger. Considering future translation to flow electrolysis, it would be beneficial to trigger these reactions using a single current, truly galvanostatic reaction configuration. With this in mind the reaction was repeated with *I*_app_ = −2.0 mA leading to 16% conversion after the 30 min electrolysis period reaching 95% after a further 120 min stirring in the absence of electrolysis (Fig. S25†). This is very promising for intensification to flow electrolysis, though it should be noted that under these conditions, a higher overall charge is passed during the reaction which has an effect on the outcome of polymerization. Whilst the control respect to the dispersity is retained (*Đ*_m_ = 1.28, Fig. S26†), the *M*_n,SEC_ (14 300 g mol^−1^) and *M*_n,th_ (6211 g mol^−1^) diverge relative the potentiostatic and step-wise current profile triggered reactions, leaving scope for optimisation in future. We attribute the divergence in the *M*_n,SEC_ and *M*_n,th_ to the gradual increase in the potential (required to maintain *I*_app_) during the initial electrolysis period. This leads to an increase in [Cu^I^(NPPI)_2_] and subsequently 
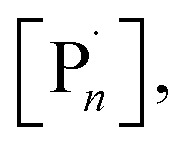
 leading to increased termination and reduced initiator efficiency, relative to the potentiostatic and step-wise current profile triggered polymerizations.

## Conclusions

In summary, seATRP using Cu(NPPI)_2_ complexes in aqueous solution has been reported for the first time. Typical electrolysis conditions require less reducing potentials (*E*_app_ = −0.08 V) than complexes derived from Me_6_-Tren and TPMA. Using OEGMA_300_ as monomer, a range of molecular weights have been targeted with the polymerizations typically complete within 2 h, yielding POEGMA_300_ with good control over the molecular weight distribution (*Đ*_m_ < 1.35). However, the defining ‘on-off’ control experiment revealed that the polymerizations were not under complete electrochemical control, as monomer conversion continued in the absence of *E*_app_. This is contrary to previous reports using more active Cu^II^L complexes. Through electrochemically triggered control experiments and UV-vis spectroscopy we have been able to propose that these less activating complexes, that stabilize Cu^I^ more than Cu^II^, follow an alternative, previously unreported, electrochemically-triggered polymerization pathway. The polymerizations proceed with good control enabling a range of molecular weights to be targeted (DP_*n*,th_ = 20–200). *In situ* chain extension is also possible alluding to potential application to the synthesis of block copolymers. The reaction set-up can also be further simplified to a 2-electrode, galvanostatic configuration which is promising for future intensification through translation to flow electrolysis. However, though suitable for eATRP at reduced catalyst loadings, more active ligands such as Me_6_-Tren, TPMA and bipy do not support the electrochemically-triggered polymerization pathway. Indeed, the ability to conduct electrochemically triggered eATRP seems to be directly related to the activity of the Cu-complex and can be related to the *k*_act_ and *K*_ATRP_ of the complexes employed. Thus, it is possible that other ligands that stabilize Cu^I^ over Cu^II^ (*e.g.* other substituted NAPI and 1,4-diazabutadiene ligands) could also follow or favour this electrochemically triggered pathway.

## Data availability

Experimental procedures and data supporting the research, not presented in the main manuscript, is included in the ESI.† Raw data files are available from the Warwick Research Archive Portal (WRAP, https://wrap.warwick.ac.uk) and from the corresponding author on request.

## Author contributions

Boyu Zhao: investigation; methodology; formal Analysis; validation; visualization; writing – original draft. Fred Pashley-Johnson: investigation; methodology; formal analysis; validation; visualization; writing – original draft. Bryn Jones: Methodology; formal analysis; supervision; writing – review and editing. Paul Wilson: conceptualization; funding acquisition; investigation; methodology; project administration; resources; supervision; writing – review and editing.

## Conflicts of interest

There are no conflicts to declare.

## Supplementary Material

SC-013-D2SC01832B-s001
