# Supplementary material for: Aqueous electrochemically-triggered atom transfer radical polymerization
Source: Chem Sci. 2022 Apr 27;13(19):5741–9. doi: 10.1039/d2sc01832b (PMC9116290; doi:10.1039/d2sc01832b)
Supplement: SC-013-D2SC01832B-s001 [file SC-013-D2SC01832B-s001.pdf]

Electronic Supplementary Material (ESI) for Chemical Science.
This journal is © The Royal Society of Chemistry 2022

Supporting Information

Aqueous Electrochemically-Triggered Atom Transfer Radical Polymerization

Boyu Zhao, Fred Pashley-Johnson, Bryn Jones and Paul Wilson*

University of Warwick, Department of Chemistry, Library Road, Coventry, UK.

E-mail: p.wilson.1@warwick.ac.uk

Experimental

1. Materials

Copper(II) bromide ($Cu^{II}Br_2$, Sigma-Aldrich, 99%), copper(I) bromide ($Cu^{I}Br$, Sigma-Aldrich, 99%), potassium chloride (KCl, Sigma-Aldrich, 99%), 2-pyridine carboxaldehyde (Aldrich, 99%), 2-picolylamine (Aldrich, 99%) sodium triacetoxyborohydride (Acros Organics, 97%), ethylene glycol, α-bromoisobutyryl bromide (Sigma Aldrich, 98%), triethylamine (Fisher Scientific, Lab grade), poly(ethylene glycol) methyl ether methacrylate (M_n = 300, 500, 1100 $g.mol^{-1}$, Sigma-Aldrich, ≥99%, with 100 ppm MEHQ as inhibitor, 300 ppm BHT as inhibitor) and tetraethylammonium tetrafluoroborate (Et_4NBF_4, AlfaAesar, 99%) were used as received without any further purification. All aqueous solutions were prepared using deionised water (15.6 MΩ, VEOLIA Elga Purelab). *N*-propyl(2-pyridyl)methanimine (NPPI), *tris*[2-(dimethylamino)ethyl]amine (Me_6TREN),[1] *tris*(2-pyridylmethyl)amine (TPMA)[2] and 2-hydroxyethyl 2-bromoisobutyrate (HEBiB)[3] were synthesised according to literature procedure and obtained with high spectroscopic purity. $Cu^{I}Br$ was purified by consecutive washes with glacial Acetic Acid (Sigma-Aldrich) and ethanol absolute (Sigma-Aldrich) to until the green colour was removed. An off white solid finally obtained after the final filtration and wash with ethanol.

2. Instruments

1H Nuclear Magnetic Resonance. 1H NMR spectra were recorded on Bruker HD-300 spectrometer utilising D_2O (Sigma-Aldrich) as solvent. Spectra were referenced and resulting chemical shifts are given in ppm relative to the residual solvent peak (δ = 4.75 ppm).

Size Exclusion Chromatography. SEC measurements were carried out using THF as the eluent with an Agilent 390-LC MDS instrument equipped with differential refractive index (DRI), viscometry (VS), dual angle light scatter (LS) and dual wavelength UV detectors. The system was equipped with 2 x PLgel Mixed C columns (300 x 7.5 mm) and a PLgel 5 µm guard column. The eluent was THF with 2 % TEA (triethylamine) and 0.01 % BHT (butylated hydroxytoluene) additives. Samples were run at 1 mL / min at 30 °C. Poly(methyl methacrylate) standards (Agilent EasyVials) were used to create a third order calibration between 550 $gmol^{-1}$ and 1,568,000 $gmol^{-1}$. Analytical samples were filtered through a GVHP membrane with 0.22 µm pore size before injection. Experimental molar mass ($M_{n,SEC}$) and dispersity ($Đ_m$) values of synthesized polymers were determined by conventional calibration using Agilent GPC/SEC software (version A.02.01).

UV-Vis spectroscopy. UV-Vis spectra were recorded on an Agilent Technologies Cary 60 UV-Vis spectrometer in the range of 250-1050 nm using a quartz cuvette (purchased from Starna) with 10 mm optical length. The $Cu^I(NPPI)_2$ calibration samples were prepared from a stock solution (10 mM) containing purified Cu^IBr (12.6 mg, 87.8 µmol, 1 eq.) and NPPI ligand (32.5 mg, 219.5 µmol, 2.5 eq.) in 9 mL DI water and Et_4NBF_4 (217 mg, 1 mmol) was added for consistency with reaction samples. Baseline measurements were recorded in DI water containing Et_4NBF_4 (217 mg, 1 mmol)

Cyclic Voltammetry (CV). Cyclic voltammetry was conducted on a CH-Instruments 600 E potentiostat using a 3 mm glassy carbon disc electrode which was polished with 0.05 µm alumina powder, rinsed sequentially with acetone, ethanol and MilliQ water prior to each use. The counter electrode was a platinum wire coil. The reference electrode

was Ag/AgCl, the silver wire was polished and rinsed sequentially with acetone, ethanol and MilliQ water the wire was then placed into a glass capillary tube fitted with a vycor frit and filled 3 M KCl solution. The supporting electrolyte Et_4NBF_4 (0.217 g, 1 mmol) and DI water (10 mL) were mixed and used as background electrolyte. A background CV was recorded to confirm the absence of impurities and oxygen. All measurements were made at a concentration of 0.0088 M with respect to $Cu^{II}L$, using $Cu^{II}Br_2$ (1 eq) as the Cu^{II} salt and NPPI (2.5 eq), TPMA (1.25 eq) and Me_6-Tren (1.25 eq) as the ligands. A CV of the catalyst system only was recorded to measure its standard reduction potential (E^θ) in pure water. To determine the E^θ in presence of monomer $OEGMA_{300}$ the process was repeated in the presence of varying amounts of $OEGMA_{300}$. Finally, HEBiB was added to evaluate the redox activity and activation behaviour of each $Cu^{I}L$ complex.

3. Experimental procedures

3.1 IKA Electrasyn reaction set-up for seATRP

The IKA electrochemical cell is composed of an undivided reaction vial and an electrode head to which working, counter and reference electrodes can be attached. For seATRP reactions an IKA manufactured Pt-coated electrode was used as the working electrode (cathode) and an IKA manufacture Ag/AgCl (using 3 M KCl) electrode was used as reference electrode. For the counter electrode, a sacrificial anode in the case of seATRP, aluminium wire (Alfa-Aesar, length = 15 cm, diameter = 1.0 mm, annealed) was manipulated to a size comparable to the IKA Pt-coated electrode and affixed into the head of the IKA electrochemical cell to serve as the counter electrode for electrolysis as reported in our previous work.[4]

3.2 General procedure for seATRP under potentiostatic conditions

In an IKA reaction vial, triethylammonium tetrafluoroborate (Et_4NBF_4, 0.217 g, 1 mmol) was dissolved in H_2O (9 mL). $CuBr_2$ (19.6 mg, 0.088 mmol), NPPI (35 µL, 0.22 mmol) were added to the solution of electrolyte and allowed to dissolve before addition of $OEGMA_{300}$ (1 mL, 3.5 mmol, 10 % v/v). HEBiB (0.175 mmol, for [M] : [I] =

[20] : [1]). The vial was then fitted with the IKA electrode head, equipped with the Pt-coated working electrode (cathode), the Ag/AgCl (using 3 M KCl) reference electrode and the aluminium counter electrode (sacrifice anode). The Electrosyn was programmed to perform electrolysis in potentiostatic mode with E_{app} = -0.16 V (this was varied depending on desired E_{app}) for a given period of time (t = 2 hr) with a stirring rate of 450 rpm (Fig. S1) at room temperature. Current vs time plots were automatically collected and stored on the IKA mobile app. Reactions were sampled periodically for reaction monitoring by ^{1}H NMR (D_2O) and SEC (THF).

3.3 Procedure for triggered seATRP under potentiostatic conditions

In an IKA reaction vial, triethylammonium tetrafluoroborate (Et_4NBF_4, 0.217 g, 1 mmol) was dissolved in H_2O (9 mL). $CuBr_2$ (19.6 mg, 0.088 mmol), NPPI (35 µL, 0.22 mmol) were added to the solution of electrolyte and allowed to dissolve before addition of $OEGMA_{300}$ (1 mL, 3.5 mmol, 10 % v/v). HEBiB (0.175 mmol, for [M] : [I] = [20] : [1]). The vial was then fitted with the IKA electrode head, equipped with the Pt-coated working electrode (cathode), the Ag/AgCl (using 3 M KCl) reference electrode and the aluminium counter electrode (sacrifice anode). The Electrosyn was programmed to perform electrolysis in potentiostatic mode with E_{app} = -0.08 V for a given period of time; t_{Eapp} = 5, 10, 20, 30 min, with a stirring rate of 450 rpm. After t_{Eapp} had elapsed the applied potential was removed (E_{app} = 0 V), a sample was taken immediately for ^{1}NMR and UV-vis analysis, then stirring was continued at 450 rpm at room temperature. Reactions were sampled periodically for reaction monitoring by ^{1}H NMR (D_2O) and UV-vis analysis.

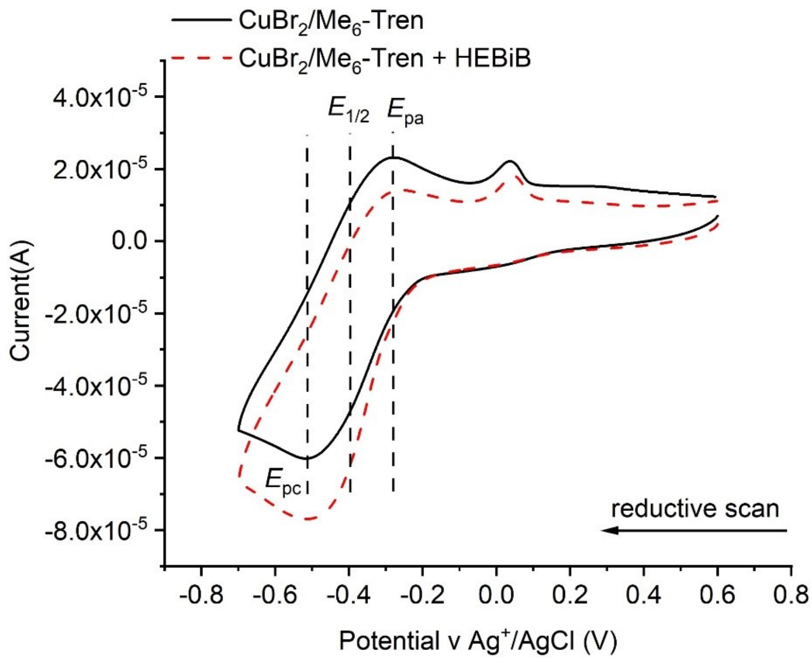

Fig S1. Cyclic voltammetry for $Cu^{II}Me_6Tren$ (8.8 mM) in $H_2O/OEGMA_{300}$ (9 : 1 v/v) + 0.1 M Et_4NBF_4 in the absence (black line) and presence (dashed red line) of 2-hydroxyethyl 2-bromoisobutyrate (HEBiB). $E_{1/2}$ = -0.40 V; $E_{p,c}$ = -0.51 V; $E_{p,a}$ = -0.28 V. Recorded on a GC electrode at v = 0.1 V.s^{-1}, room temperature.

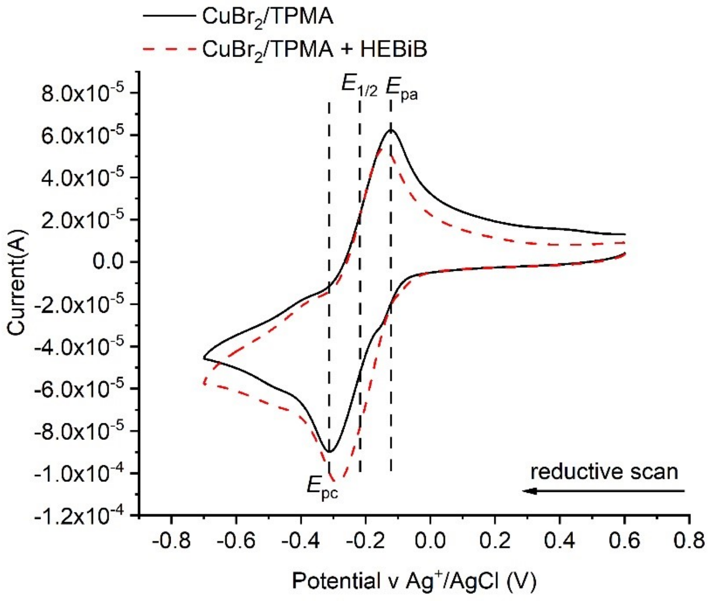

Fig S2. Cyclic voltammetry for $Cu^{II}TPMA$ (8.8 mM) in $H_2O/OEGMA_{300}$ (9 : 1 v/v) + 0.1 M Et_4NBF_4 in the absence (black line) and presence (dashed red line) of 2-hydroxyethyl 2-bromoisobutyrate (HEBiB). $E_{1/2}$ = -0.21 V; $E_{p,c}$ = -0.31 V; $E_{p,a}$ = -0.12 V. Recorded on a GC electrode at v = 0.1 V.s^{-1}, room temperature.

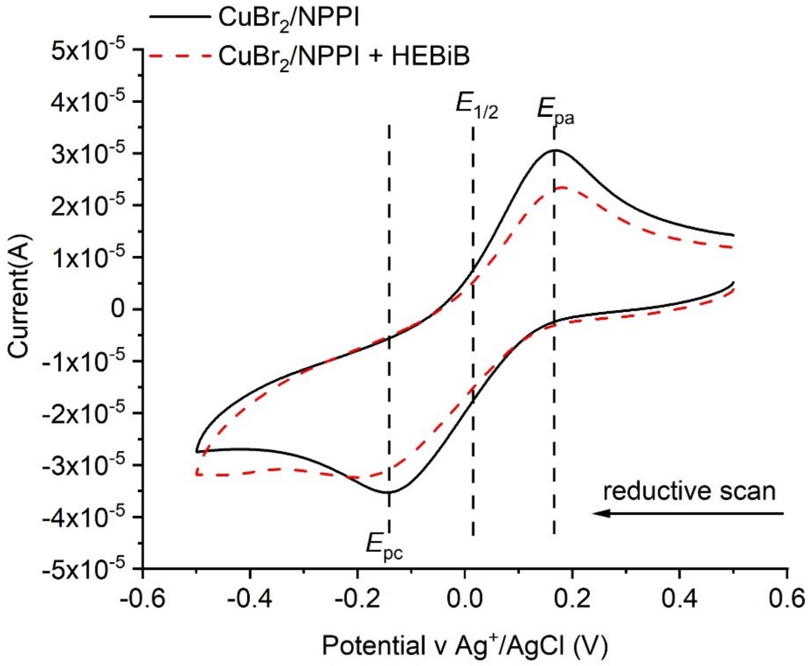

Fig S3. Cyclic voltammetry for $Cu^{II}(NPPI)_2$ (8.8 mM) in H_2O/$OEGMA_{300}$ (9 : 1 v/v) + 0.1 M Et_4NBF_4 in the absence (black line) and presence (dashed red line) of 2-hydroxyethyl 2-bromoisobutyrate (HEBiB). $E_{1/2}$ = 0.02 V; $E_{p,c}$ = -0.14 V; $E_{p,a}$ = 0.17 V. Recorded on a GC electrode at v = 0.1 V.s^{-1}, room temperature.

Table S1. seATRP of $OEGMA_{300}$ (10% v/v, unless stated otherwise) in H_2O. [$OEGMA_{300}$] : [HEBiB] = [20] : [1]

Entry	Ligand	[$Cu^{II}Br_2$] : [L]	E_{app} / V	Time / h	Conv[a]	$M_{n,th}$[b] / g.mol^{-1}	$M_{n,SEC}$[c] / g.mol^{-1}	$Đ_m$[c]
1	NPPI	[0.5] : [1.25]	-0.04	2	100%	6211	9200	1.31
2	Me_6Tren	[0.5] : [0.6]	-0.4	4	70%	4348	21200	5.05
3	TPMA	[0.5] : [0.6]	-0.2	4	80%	4969	5400	4.24

[a] Determined via ^{1}H NMR of reaction samples performed in D_2O. [b] $M_{n,th}$ = [(conv./100 x $DP_{n,th}$) x 300] + 211 [c] From THF SEC of purified polymers.

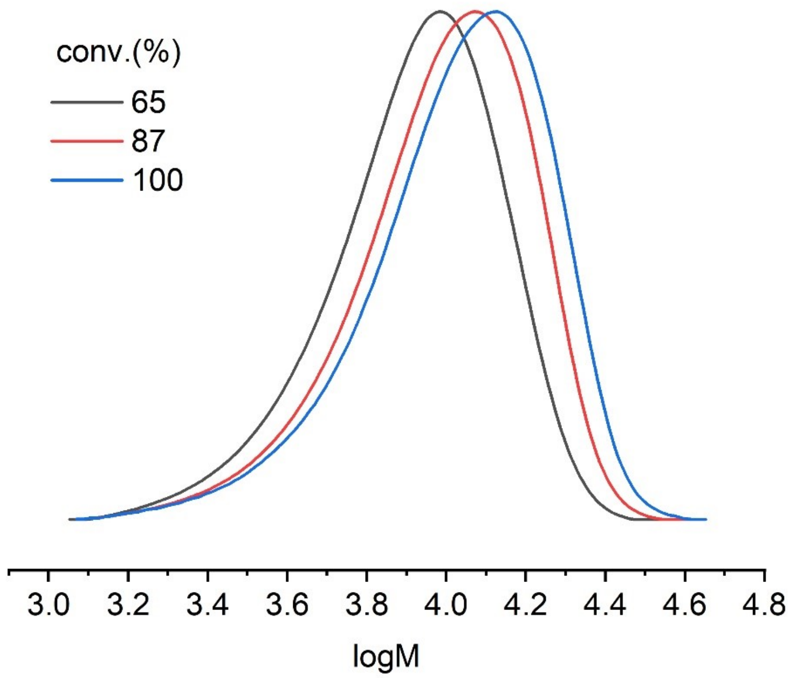

Fig S4. SEC in THF of reaction samples taken during the potentiostatic (E_{app} = -0.04 V) seATRP of $OEGMA_{300}$ (10% v/v) in H_2O ([$OEGMA_{300}$] : [HEBiB] : [$CuBr_2$] : [NPPI] = [20] : [1] : [0.5] : [1.25]); $M_{n,SEC}$ = 9200 g.mol^{-1} and $Đ_m$ = 1.31 (blue trace).

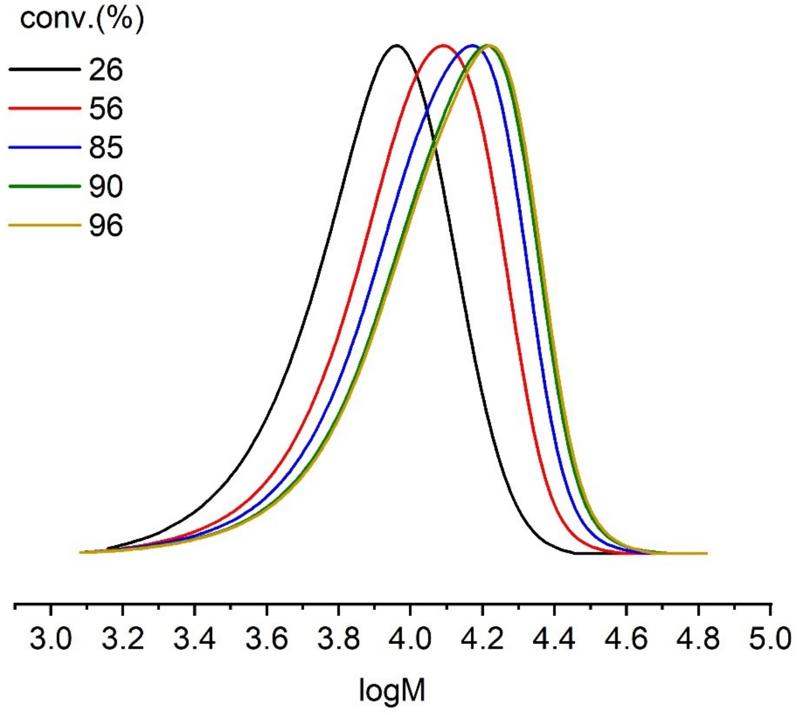

Fig S5. SEC in THF of reaction samples taken during the potentiostatic (E_{app} = -0.08 V) seATRP of $OEGMA_{300}$ (10% v/v) in H_2O ([$OEGMA_{300}$] : [HEBiB] : [$CuBr_2$] : [NPPI] = [20] : [1] : [0.5] : [1.25]); $M_{n,SEC}$ = 11000 g.mol^{-1} and $Đ_m$ = 1.30 (gold trace).

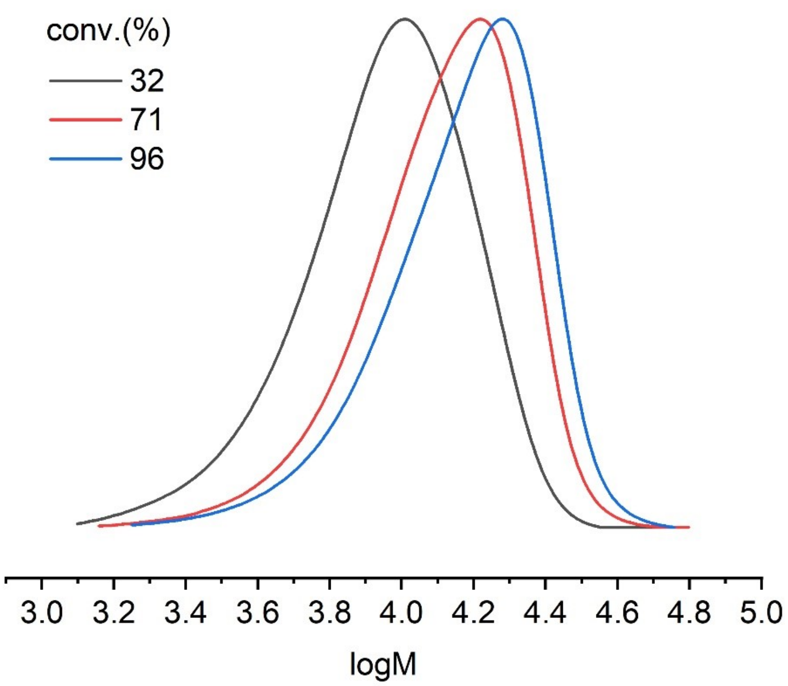

Fig S6. SEC in THF of reaction samples taken during the potentiostatic (E_{app} = -0.12 V) seATRP of $OEGMA_{300}$ (10% v/v) in H_2O ([$OEGMA_{300}$] : [HEBiB] : [$CuBr_2$] : [NPPI] = [20] : [1] : [0.5] : [1.25]); $M_{n,SEC}$ = 12800 g.mol^{-1} and $Đ_m$ = 1.29 (blue trace).

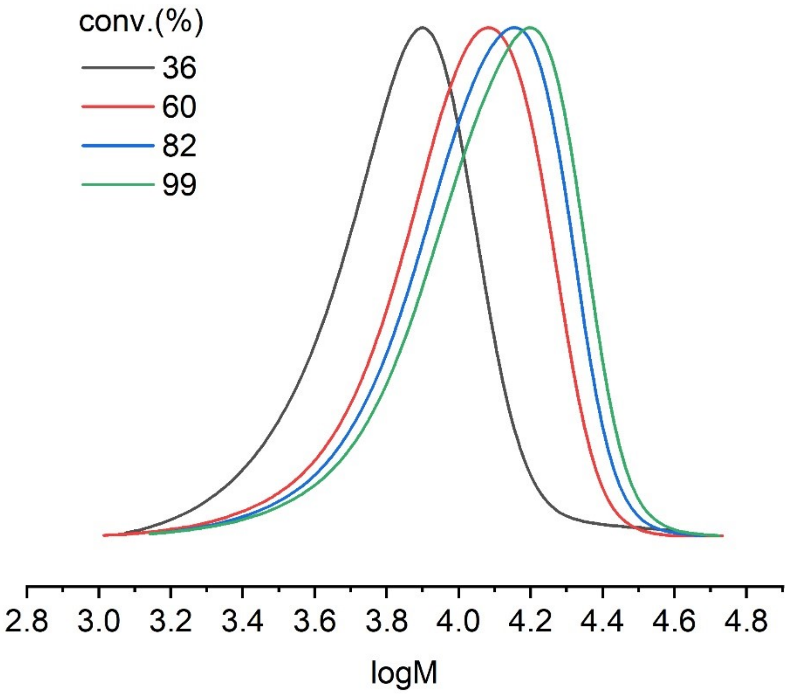

Fig S7. SEC in THF of reaction samples taken during the potentiostatic (E_{app} = -0.16 V) seATRP of $OEGMA_{300}$ (10% v/v) in H_2O ([$OEGMA_{300}$] : [HEBiB] : [$CuBr_2$] : [NPPI] = [20] : [1] : [0.5] : [1.25]); $M_{n,SEC}$ = 10200 g.mol^{-1} and $Đ_m$ = 1.32 (green trace).

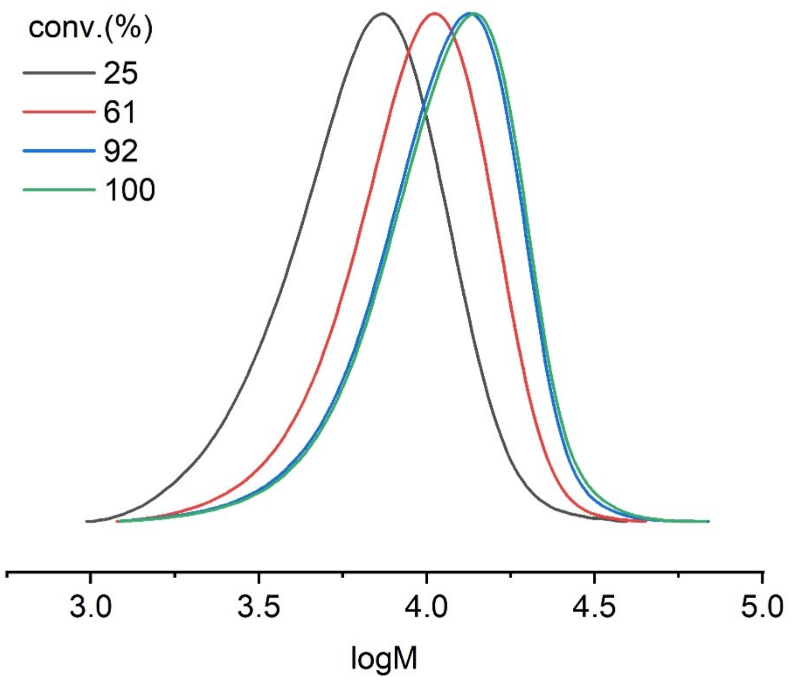

Fig S8. SEC in THF of reaction samples taken during the potentiostatic (E_{app} = -0.16 V) seATRP of $OEGMA_{300}$ (20% v/v) in H_2O ([$OEGMA_{300}$] : [HEBiB] : [$CuBr_2$] : [NPPI] = [20] : [1] : [0.5] : [1.25]); $M_{n,SEC}$ = 10000 g.mol^{-1} and $Đ_m$ = 1.28 (green trace).

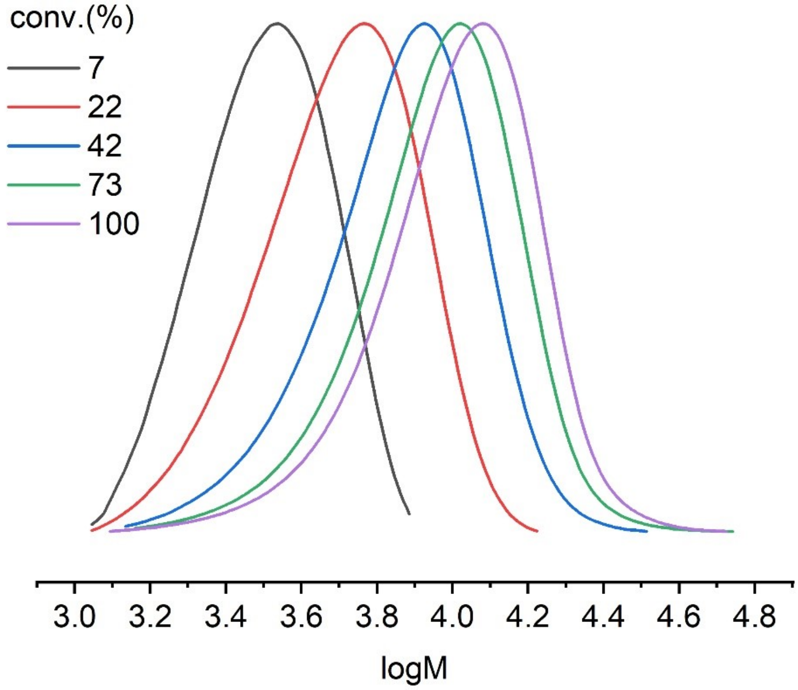

Fig S9. SEC in THF of reaction samples taken during the potentiostatic (E_{app} = -0.16 V) seATRP of $OEGMA_{300}$ (30% v/v) in H_2O ([$OEGMA_{300}$] : [HEBiB] : [$CuBr_2$] : [NPPI] = [20] : [1] : [0.5] : [1.25]); $M_{n,SEC}$ = 9000 g.mol^{-1} and $Đ_m$ = 1.26 (green trace).

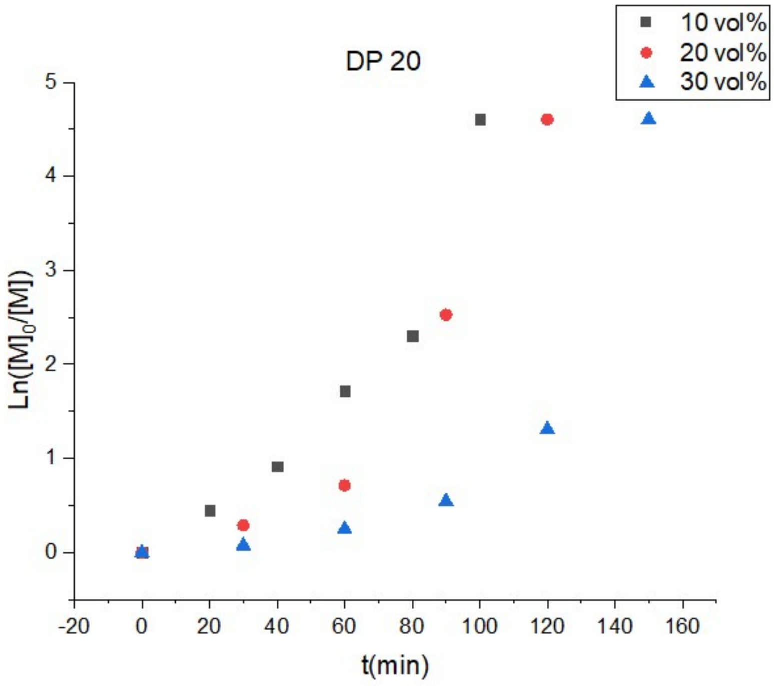

Fig S10. Kinetic analysis for [$OEGMA_{300}$] : [HEBiB] : [$CuBr_2$] : [NPPI] = [20] : [1] : [0.5] : [1.25] at 10 % (black), 20 % (red) and 30 % (blue) v/v $OEGMA_{300}$. (E_{app} = -0.16 V)

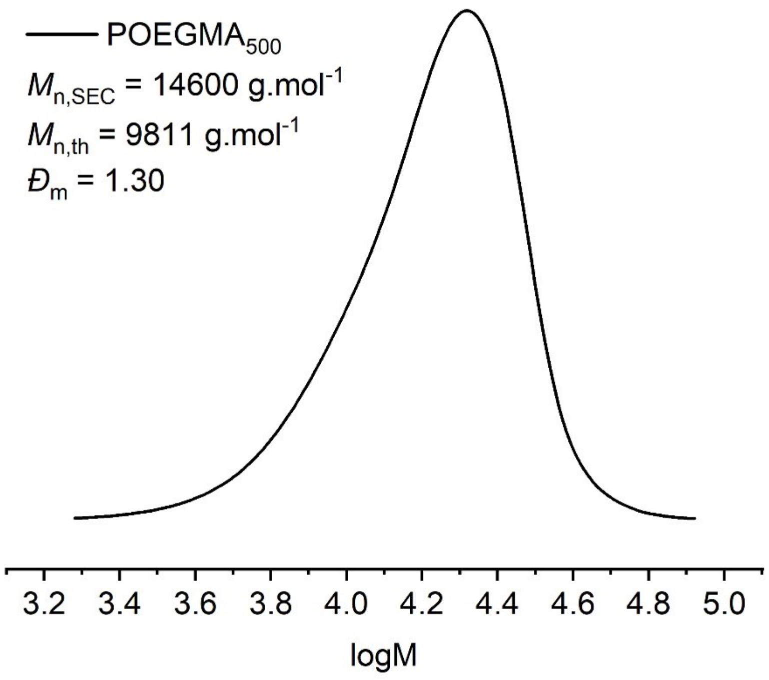

Fig S11. SEC in THF of reaction samples taken during the potentiostatic (E_{app} = -0.16 V) seATRP of $OEGMA_{500}$ (10% v/v) in H_2O ([$OEGMA_{500}$] : [HEBiB] : [$CuBr_2$] : [NPPI] = [20] : [1] : [0.5] : [1.25]).

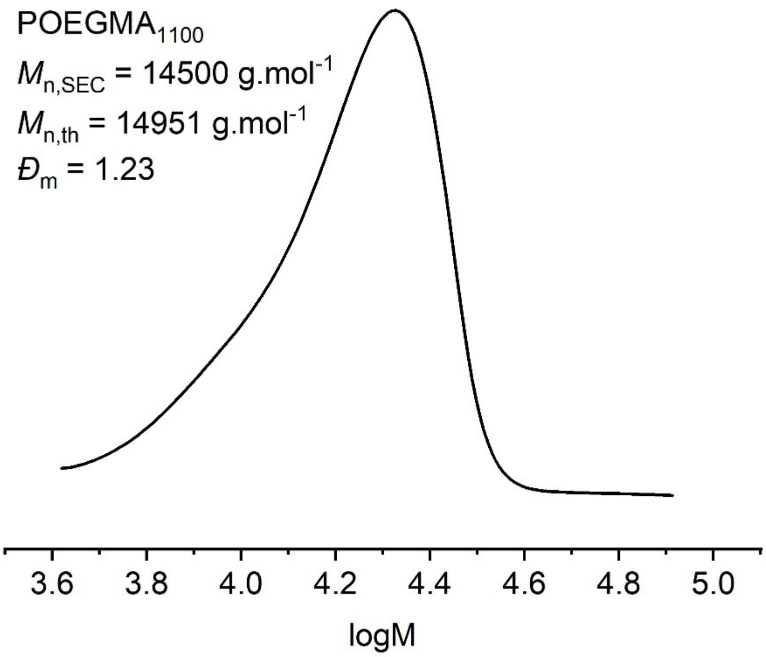

Fig S12. SEC in THF of reaction samples taken during the potentiostatic (E_{app} = -0.16 V) seATRP of $OEGMA_{1100}$ (10% v/v) in H_2O ([$OEGMA_{1100}$] : [HEBiB] : [$CuBr_2$] : [NPPI] = [20] : [1] : [0.5] : [1.25]).

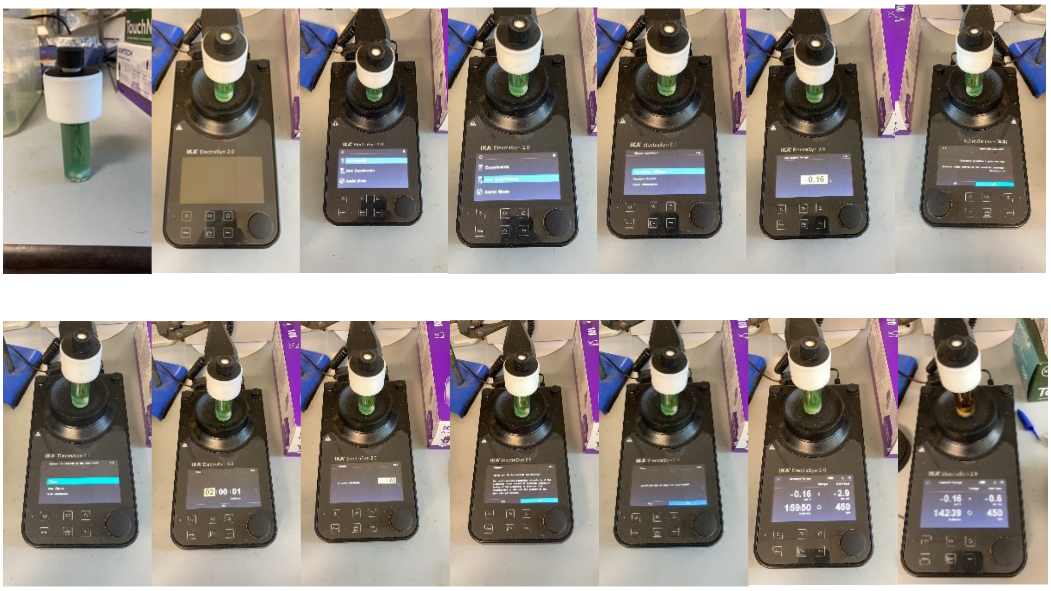

Fig S14. Example of the Electrasyn interface used to set the reaction parameters. Here a potentiostatic reaction at E_{app} = -0.16 V is demonstrated. The observed colour change from green to brown is attributed to electrochemical reduction of $Cu^{II}(NPPI)_2$ (green) to $Cu^{I}(NPPI)_2$ (brown).

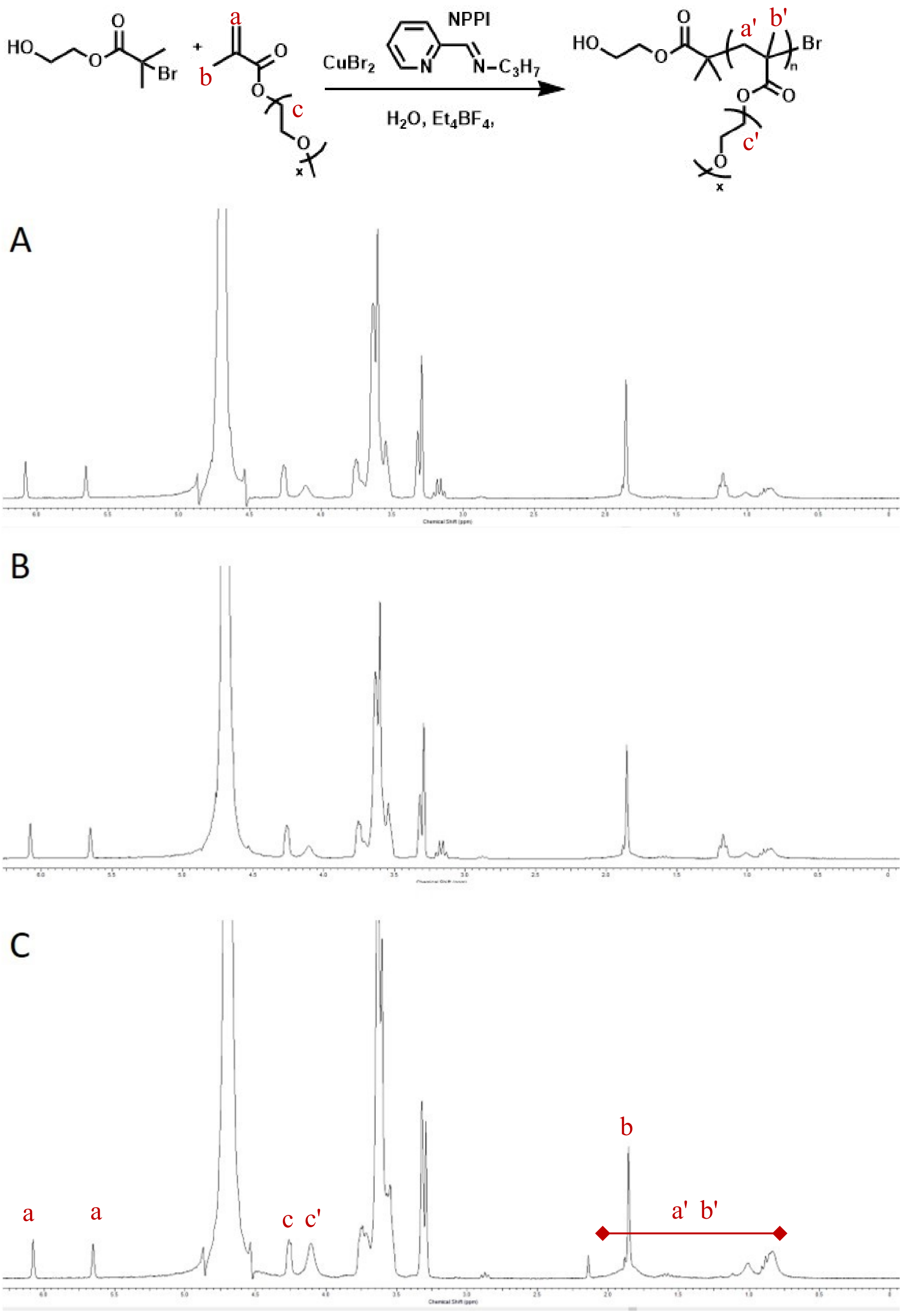

Fig S15. ^{1}H NMR for the potentiostatic (E_{app} = -0.08 V) seATRP of OEGMA$_{300}$ (10% v/v) in H_2O ([OEGMA$_{300}$] : [HEBiB] : [$CuBr_2$] : [NPPI] = [20] : [1] : [0.5] : [1.25]). (A) E_{app} = -0.08 V, t = 30 min, conversion = 33%. (B) E_{app} = 0 V with sparging with compressed air, t = 30 min, conversion = 33%. (C) N_2 sparging (15 min) then E_{app} = -0.08 V, t = 30 min, conversion = 64% (Conversions calculated from the relative integrals of H_c and H_c’).

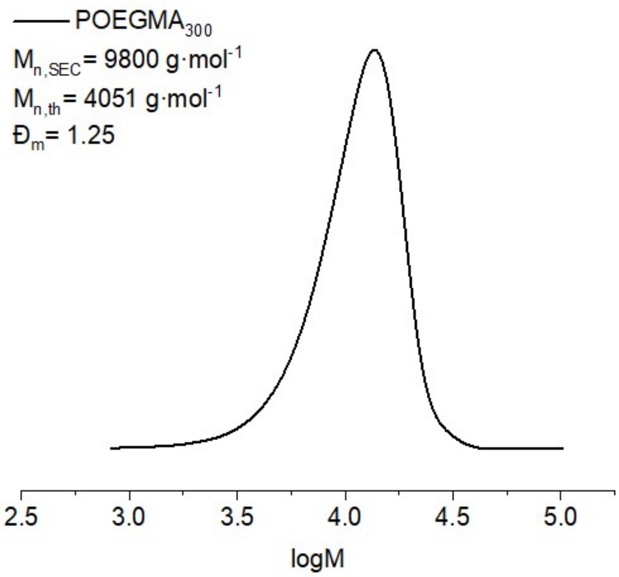

Fig S16. SEC in THF of $POEGMA_{300}$ obtained from Fig S15C.

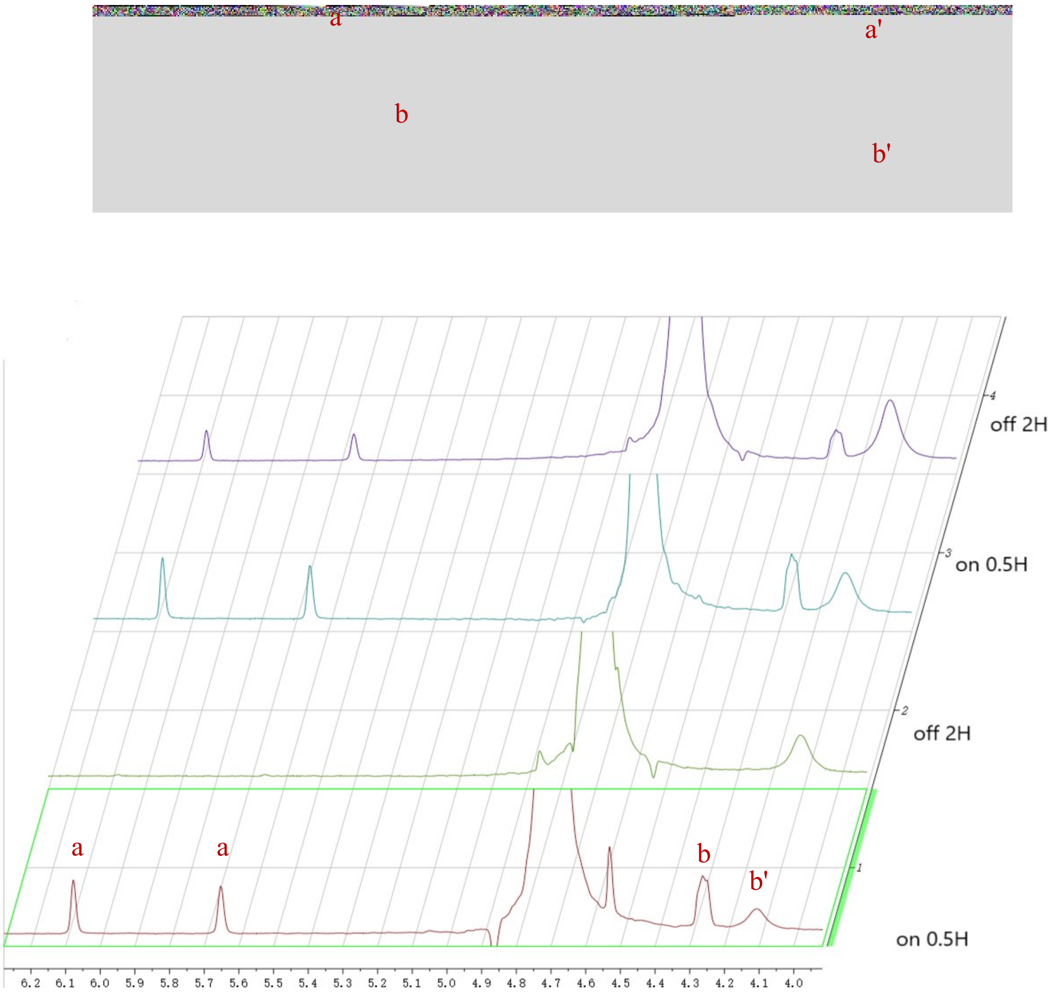

Fig S17. ^1H NMR for electrochemically triggered eATRP of $OEGMA_{300}$ ($[OEGMA_{300}]$: [HEBiB] : $[CuBr_2]$: [NPPI] = [20] : [1] : [0.5] : [1.25]; E_{app} = -0.08 V; t_{Eapp} = 30 min) and chain extension ($[OEGMA_{300}]$: $[POEGMA_{300}\text{-}Br]$ = [20] : [1]; E_{app} = -0.08 V; t_{Eapp} = 30 min) (Conversions reported in Table 2 were calculated from the relative integrals of H_b and H_b’).

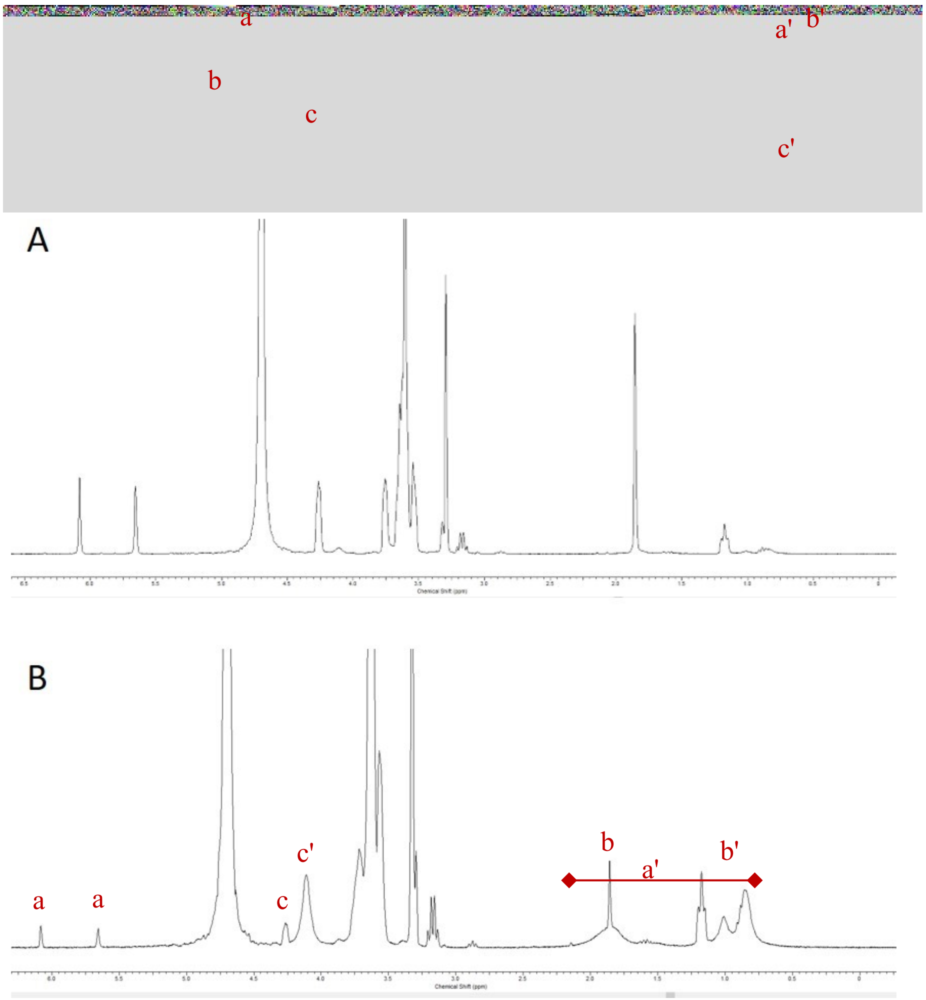

Fig S18. ^{1}H NMR for the potentiostatic (E_{app} = -0.08 V) seATRP of $OEGMA_{300}$ (10% v/v) in H_2O ([$OEGMA_{300}$] : [HEBiB] : [$CuBr_2$] : [NPPI] = [200] : [1] : [0.5] : [1.25]). (A) E_{app} = -0.08 V, t = 30 min, conversion = 13%. (B) E_{app} = 0 V, t = 90 min, conversion = 89% (Conversions calculated from the relative integrals of H_c and H_c’).

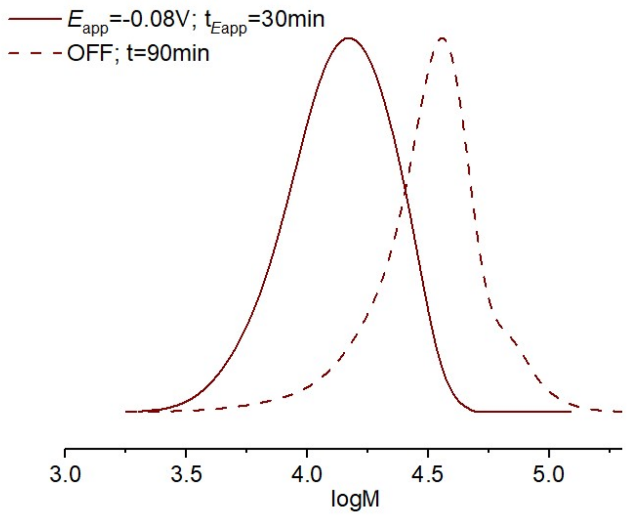

Fig S19. SEC in THF for the potentiostatic (E_{app} = -0.08 V) seATRP of $OEGMA_{300}$ (10% v/v) in H_2O ([$OEGMA_{300}$] : [HEBiB] : [$CuBr_2$] : [NPPI] = [200] : [1] : [0.5] : [1.25]) showing the evolution of the molecular weight distribution after electrolysis (E_{app} = -0.08 V, t_{Eapp} = 30 min, solid line) and after the potential was removed (E_{app} = 0 V, dashed lines, t = 90 min) final $M_{n,SEC}$ = 27200 g.mol^{-1}, $Đ_m$ = 1.32).

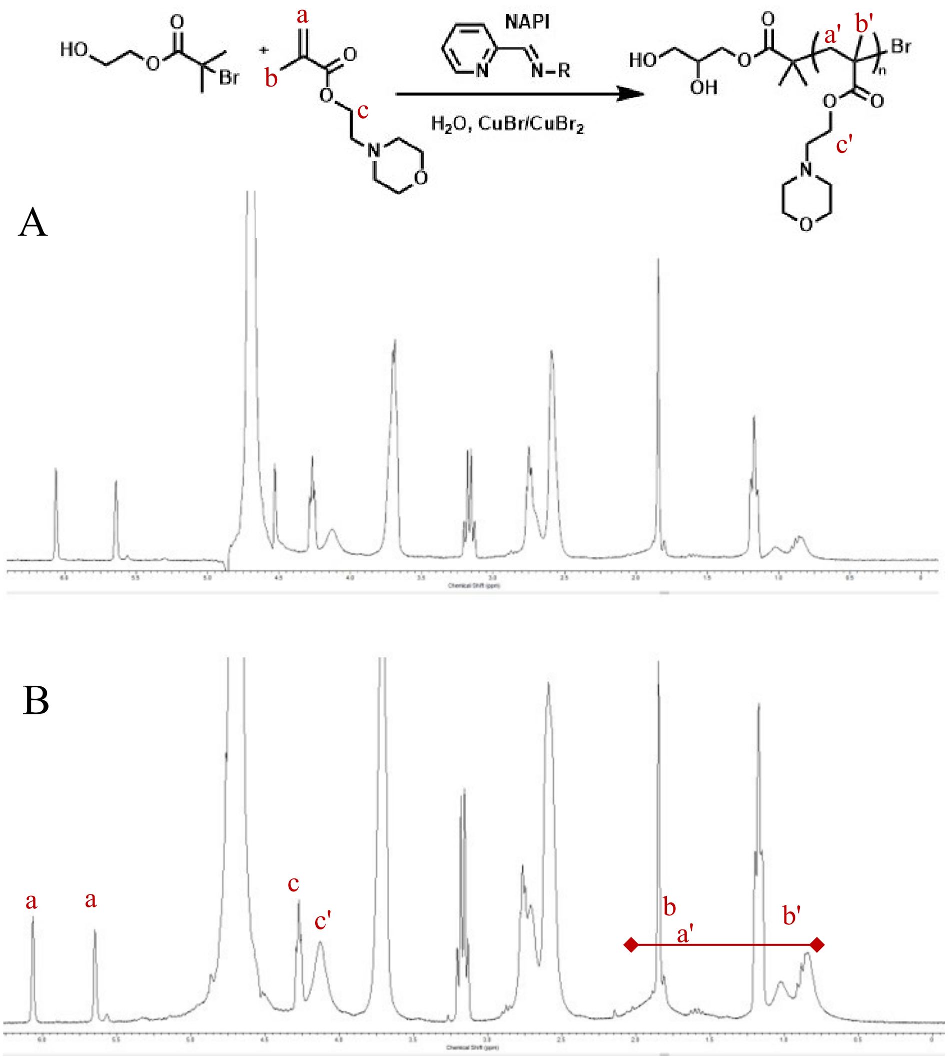

Fig S20. ^{1}H NMR for the potentiostatic (E_{app} = -0.08 V) seATRP of 2-*N*-morpholinoethyl methacrylate (10% v/v) in H_2O ([M] : [HEBiB] : [$CuBr_2$] : [NPPI] = [200] : [1] : [0.5] : [1.25]). (A) E_{app} = -0.08 V, t = 30 min, conversion = 40%. (B) E_{app} = 0 V, t = 90 min, conversion = 65% (Conversions calculated from the relative integrals of H_c and H_c’).

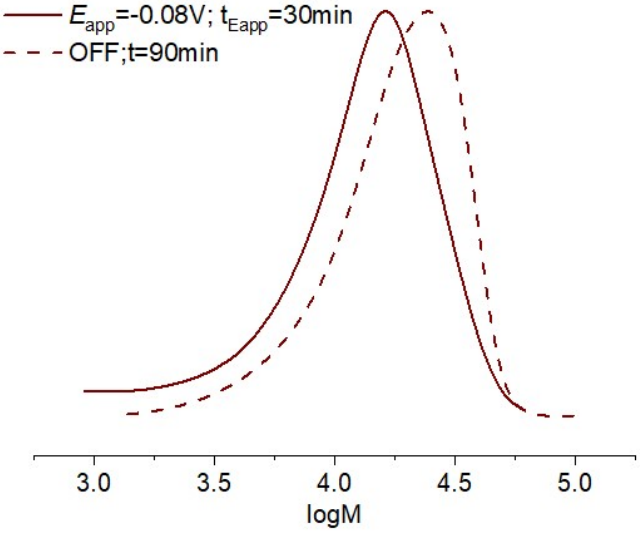

Fig S21. SEC in THF for the potentiostatic (E_{app} = -0.08 V) seATRP of 2-*N*-morpholinoethyl methacrylate (10% v/v) in H_2O ([M] : [HEBiB] : [$CuBr_2$] : [NPPI] = [200] : [1] : [0.5] : [1.25]) showing the evolution of the molecular weight distribution after electrolysis (E_{app} = -0.08 V, t_{Eapp} = 30 min, solid line) and after the potential was removed (E_{app} = 0 V, dashed lines, t = 90 min) final $M_{n,SEC}$ = 14000 g.mol^{-1}, $Đ_m$ = 1.47).

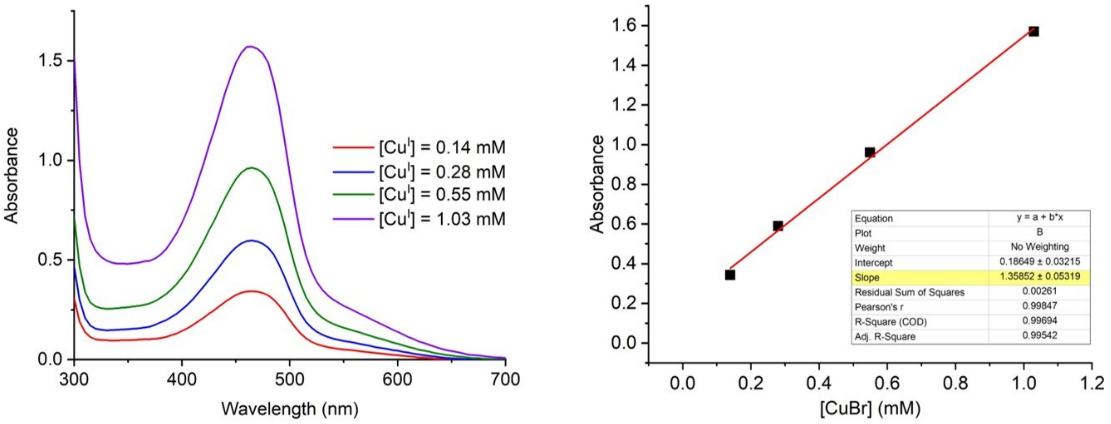

Fig S22. UV-vis calibration data to determine the molar extinction coefficient (ε) for $Cu^I(NPPI)_2$. Samples were prepared from a stock solution of $Cu^I(NPPI)_2$ (10 mM) which was diluted in order to obtain a maximum absorbance close to 1 (left). Serial dilution allowed a calibration plot to be constructed (right) from which ε = 1359 $M^{-1}.cm^{-1}$. For the following reactions this was used to quantify [$Cu^I(NPPI)_2$] using the Beer-Lambert Law A = ε.c.l.

Determination of [$Cu^I(NPPI)_2$] in triggered seATRP reactions

Triggered seATRP reactions were performed using [$OEGMA_{300}$] : [HEBiB] : [$CuBr_2$] : [NPPI] = [20] : [1] : [0.5] : [1.25], E_{app} = -0.08 V and t_{Eapp} = 30 mins. Undiluted reaction samples gave absorbance values at λ = 464 nm > 5 A.U. Consequently, 0.5 mL of the reaction solution was taken and diluted with 2.5 mL DI water i.e. 6x dilution. This gave a maximum absorbance close to 1 A.U. [$Cu^I(NPPI)_2$] in mM was determined using the Beer-Lambert Law c = (A / ε.l) x 6. Where c = [$Cu^I(NPPI)_2$], A = measure absorbance (at 6x dilution), ε = molar extinction coefficient of $Cu^I(NPPI)_2$ (1359 $M^{-1}.cm^{-1}$ = 1.36 $mM^{-1}.cm^{-1}$), l = pathlength (1 cm) and X 6 accounts for the dilution factor applied.

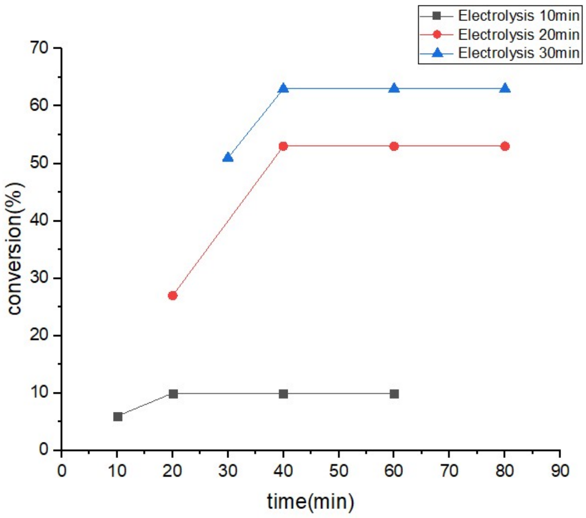

Fig S23. Conversion vs time plot for the triggered seATRP of [$OEGMA_{300}$] : [HEBiB] : [$CuBr_2$] : [bipy] = [20] : [1] : [0.5] : [1.25]. E_{app} = -0.08 V, t_{Eapp} = 10 min (black), 20 min (red), 30 min (blue).

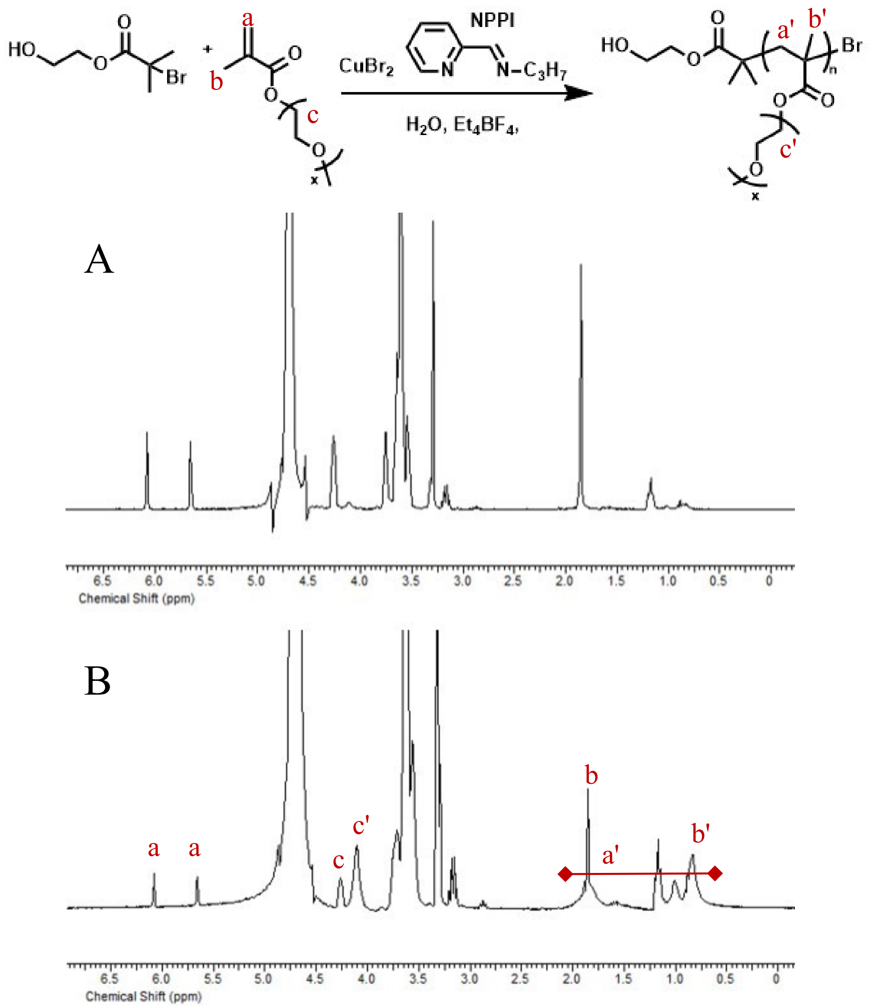

Fig S24. ^{1}H NMR for the seATRP of $OEGMA_{300}$ (10% v/v) in H_2O ([$OEGMA_{300}$] : [HEBiB] : [$CuBr_2$] : [NPPI] = [20] : [1] : [0.5] : [1.25]) using a step-wise current profile. (A) I_{app} = -3.5 mA, 8 min; -1.9 mA, 7 min; -0.5 mA, 15 min, conversion = 12% (Q = 2.93 C). (B) I_{app} = 0 mA, t = 120 min, conversion = 80%. (Conversions calculated from the relative integrals of H_c and H_{c}').

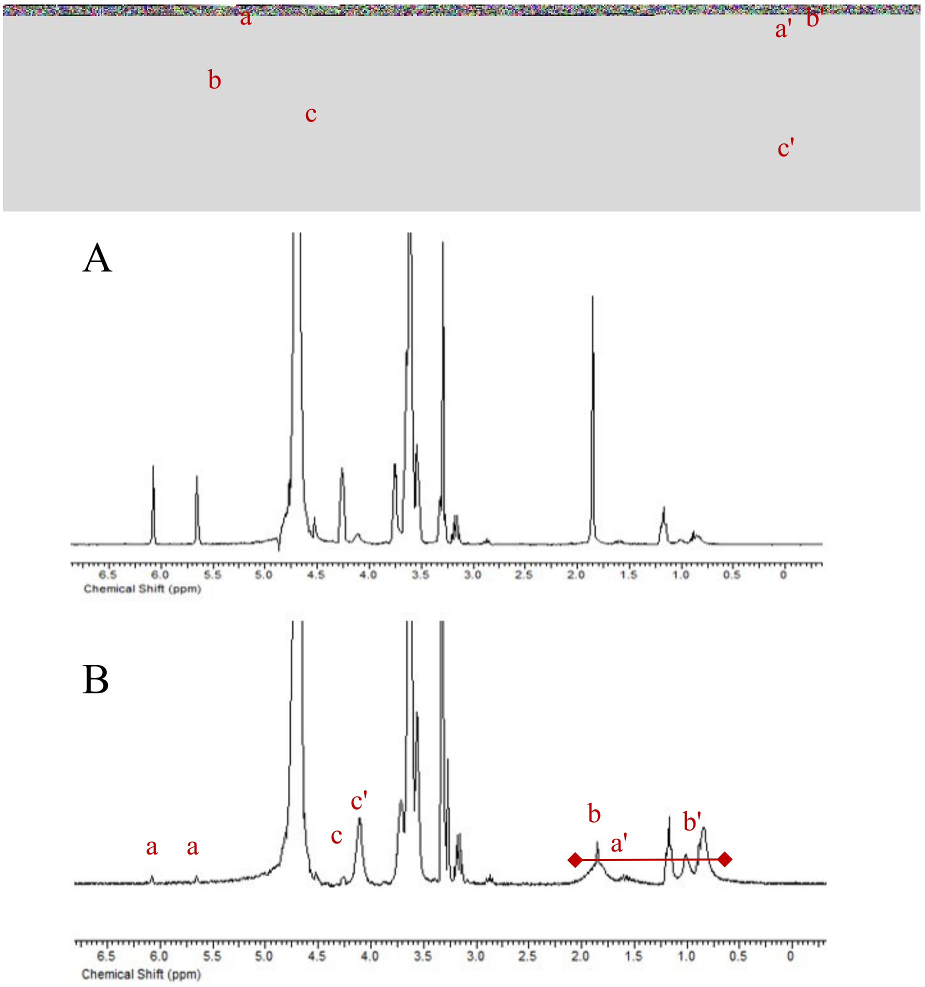

Fig S25. ^{1}H NMR for the seATRP of $OEGMA_{300}$ (10% v/v) in H_2O ([$OEGMA_{300}$] : [HEBiB] : [$CuBr_2$] : [NPPI] = [20] : [1] : [0.5] : [1.25]) using a step-wise current profile. (A) I_{app} = -2.0 mA, t = 30 min, conversion = 16% (Q = 3.6 C). (B) I_{app} = 0 mA, t = 120 min, conversion = 95%. (Conversions calculated from the relative integrals of H_c and H_c').

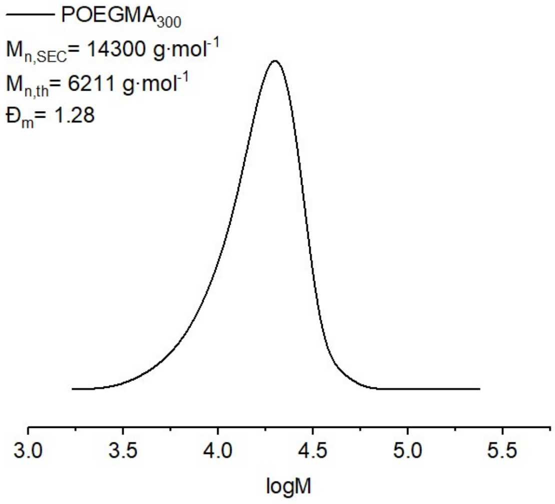

Fig S26. SEC in THF for the galvanostatic (I_{app} = -2.0 mA, Fig S25B) seATRP of $OEGMA_{300}$ (10% v/v) in H_2O ([$OEGMA_{300}$] : [HEBiB] : [$CuBr_2$] : [NPPI] = [20] : [1] : [0.5] : [1.25]).

References

1. a) Ciampolini, M.; Nardi, N. *Inorg. Chem.* **1966**, *5*, 41; b) Queffelec, J.; Gaynor, S. G.; Matyjaszewski, K. *Macromolecules* **2000**, *33*, 8629

2. Beni, A.; Dei, A.; Laschi, S.; Rizzitano, M.; Sorace, L. *Chem. Eur. J.* **2008**, *14*, 1804-1813.

3. Edwards, S. E.; Flynn, S.; Hobson, J. J.; Chambon, P.; Cauldbeck, H.; Rannard, S. P. *RSC Adv.* **2020**, *10*, 30463-30475.

4. Zhao, M. Mohammed, B. A. Jones, P. Wilson, *Chem. Commun.* **2021***, 57,* 3897-3900
